# A high throughput ambient mass spectrometric approach for identifying the poaching of wild american ginseng

**DOI:** 10.1186/s40068-025-00414-6

**Published:** 2025-12-20

**Authors:** Julia E. Shaw, Pamela Brunswick, Jolene Lesuk, Lola Rabinovitch, Taylor Filewood, Honoria Kwok, Jeffrey Yan, Robert Cody, Tao Huan, Dayue Shang

**Affiliations:** 1 Department of Chemistry, Faculty of Science, University of British Columbia, 2036 Main Mall, Vancouver, BC V6T 1Z1 Canada; 2 Science and Technology Branch, Pacific Environmental Science Centre, Environment and Climate Change Canada, Pacific and Yukon Laboratory for Environmental Testing, North Vancouver, BC V7H 1V2 Canada; 3 JEOL USA Inc, Peabody, MA USA

**Keywords:** Wild American ginseng, Species identification, Direct analysis in real time, *Panax quinquefolius*, Multivariate statistics

## Abstract

Rapid identification is critically important in the protection of endangered species listed under the Convention on International Trade in Endangered Species (CITES). One such species is American ginseng (*Panax quinquefolius*), whose remaining wild populations are vulnerable to the effects of poaching. Direct Analysis in Real Time Time-of-Flight Mass Spectrometry (DART-ToF MS) is a mature but underutilized forensic tool suitable for rapidly analyzing plant materials. This tool offers greater convenience over alternative species identification methods commonly requiring extensive sample preparation and instrument run times. In the current study, four categories of ginseng, including wild and cultivated American ginseng, Korean ginseng (*P. ginseng*), and Chinese ginseng (*P. notoginseng*), were analyzed by DART-ToF MS. The collected mass spectra were visually compared by heat map prior to application of multivariate statistical analysis to cluster sample groups, yielding a two-step identification model capable of identifying the origin of blind quality assurance samples. With fast sample preparation, data acquisition, and statistical analysis, DART-ToF MS shows great potential as a forensic screening tool in combating poaching and illegal trade of endangered and CITES-listed species such as wild American ginseng.

**Supplementary Information:**

The online version contains supplementary material available at 10.1186/s40068-025-00414-6.

## Introduction

Ginseng, a herb commonly employed in traditional medicines (TM), is renowned for its pharmaceutical capabilities (Court 2000). The three commercially relevant species of ginseng, namely Chinese ginseng (*Panax notoginseng*), Korean ginseng (*Panax ginseng*), and American ginseng (*Panax quinquefolius*), are considered to have unique medicinal properties (Court 2000). Korean ginseng is regarded to have a “warming” effect on the consumer, possessing anti-tumor properties, improving immunity, and treating some inflammatory diseases (Court 2000; Yang et al. 2016). Chinese ginseng, also referred to as “Sanchi” or “Sanqi”, is most notable for treating ischemia, cardiovascular diseases, pain, swelling, or other hemostatic ailments (Yang et al. 2018; Chen et al. 2013). American ginseng, found in Northeastern America, is believed to have a “cooling” effect on the user (Yang et al. 2016; Panax in Flora of China @ efloras.org 2024). Similar to Korean ginseng, American ginseng is considered to have properties useful for treating cancer, improving immunity, and reducing inflammation (Court 2000; Khan et al. 2015). The popularity of American ginseng in TM has resulted in excessive overharvesting of its wild populations, leading to the herbs inclusion in CITES Appendix II and its regulation in Canada (Panax quinquefolius L. 2024; Canada E. and C. C 2024a; American ginseng (Panax quinquefolius) 2024). Poaching of wild American ginseng is commonly fueled by traditional beliefs that suggest the wild herb is more potent than cultivated varieties, leading to increased demand (McGraw et al. 2013). Consequentially, wild herbs can be valued up to $2200 USD per kg compared to $70 USD per kg for cultivated American ginseng (Sheban et al. 2022; Shaw et al. 2025). To increase the value of their products, some American ginseng cultivators have attempted to produce wild simulated farming or “forest farming” (Sheban et al. 2022). Due to the interest of the pharmaceutical industry and cultivators, American ginseng may be considered a phytometer species as the herb’s biology has been extensively investigated (McGraw et al. 2013). Thus, the behavior and population dynamics of surviving wild American ginseng communities are informative of the general trends of species occupying similar biospheres in North America (Sheban et al. 2022).

American ginseng’s wild populations are threatened by several factors including overharvesting, deforestation, suburbanization, climate change, and deer browsing (McGraw et al. 2013). Overharvesting of American ginseng threatens the species viability by reducing the available genetic diversity in the wild populations (Cruse-Sanders and Hamrick 2004). Size selective harvest of the largest individuals in wild American ginseng populations, driven by both profit and harvesting regulations, depletes the average size, reduces growth rates, and decreases reproductive capabilities of each passing generation (Cruse-Sanders and Hamrick 2004). Ultimately, the current situations threaten the species viability, highlighting the negative impact of excessive human harvest on ginseng and other herbaceous species in North America. The vulnerability of ginseng populations in North America motivated their protection under federal and provincial, or state, laws across Canada and the USA. Internationally, the species is listed under CITES Appendix II, indicating that it is likely to be threatened by extinction if trade is not controlled (Burkhart et al. 2012). The CITES regulations demand that only mature plants, with greater than 3 leaves or 5 leaflets, may be harvested (Burkhart et al. 2012). This regulation attempts to protect the wild populations by allowing individual plants to undergo reproductive events before being harvested (Burkhart et al. 2012). Unfortunately, these restrictions further encourage size selective harvest of wild populations, exasperating the negative generational changes inflicted by such practice (Cruse-Sanders and Hamrick 2004).

In Canada, American ginseng is classified as an endangered species and protected under the Species at Risk Act on federal land, prohibiting all wild harvest (American ginseng (Panax quinquefolius) 2024). The export of wild American ginseng is illegal under the Wild Animal and Plant Protection and Regulation of International and Interprovincial Trade Act (WAPPRIITA) (American ginseng (Panax quinquefolius) 2024). This act further requires that exporters of cultivated American ginseng first obtain a CITES permit (American ginseng (Panax quinquefolius) 2024). In Ontario, the species is further protected under the Ontario Endangered Species Act 2007. (American ginseng (Panax quinquefolius) 2024) This act prohibits the harvest, possession, and sale of both wild and cultivated American ginseng with the exception of cultivated ginseng grown under license from the Ontario Ginseng Growers Association (American ginseng (Panax quinquefolius) 2024). In Québec, wild American ginseng is classified as threatened and its harvest and trade is prohibited under Loi sur les espèces menacées ou vulnérables (American ginseng (Panax quinquefolius) 2024). Those found guilty of poaching wild American ginseng in Québec can face jail time of up to 5 years or fines of $150,000 CD or $300,000 CD for individuals and businesses respectively. (American ginseng (Panax quinquefolius) 2024) While the legal infrastructure to protect the remaining wild American ginseng populations is established in North America, its enforcement is a major challenge due to the lack of easy and rapid means to identify the ginseng species and provenance. Anecdotal evidence from harvesters and cultivators alike suggest that these regulations are rarely enforced (Burkhart et al. 2012). To prosecute poachers, strong forensic evidence of illegal harvest is required. This evidence may exist on two levels of specification: species validation and provenance identification. Confirmation of species can be useful to both provide evidence that an illegal harvester is trading the CITES listed species without the required permits and identify mislabeled commercial products. The ability to identify a particular sample as wild or cultivated in origin constitutes strong evidence to implicate a poaching offence. Upholding the laws and regulation surrounding American ginseng harvest and cultivation is essential to deter poaching activities and secure the viability of the species.

Several methods of ginseng species identification have been developed with the primary objective of product authentication. Previously, ginseng species have been distinguished by morphological features. However, two of the commercially relevant species, including American and Korean ginseng, share similar morphology and are difficult to distinguish in such a manner (Sharma and Pandit 2009). Additionally, commercial ginseng products are rarely sold as whole roots and instead are typically processed into products such as teas, powders, or extracts, complicating their identification (Court 2000). Analytical methods relying on the intrinsic chemical content of ginseng specimen, including genetic analysis, protein analysis, and chemotyping, were developed to address the limitations of morphological analysis by providing evidence independent of sample form. Genetic analysis relies on the genetic diversity of ginseng species for differentiation (Lu et al. 2022). An array of methods in this area have been developed for ginseng species identification including amplified fragment-length polymorphism (AFLP) (Osathanunkul and Madesis 2019), random amplified polymorphic DNA (RAPD) (Kim et al. 2007; Shim et al. 2003), site specific PCR (Lu et al. 2022), and DNA bar coding (Lu et al. 2022; Osathanunkul and Madesis 2019; Zuo et al. 2011; Chen et al. 2023). While these methods have been successful in differentiating relevant species, this approach is limited by the quality of DNA in processed commercial samples (Lu et al. 2022). Studies investigating the protein content of ginseng species for identification typically employ sodium dodecyl sulfate–polyacrylamide gel electrophoresis (SDS-PAGE) or 2 dimensional gel electrophoresis (2DE) to separate proteins and may include mass spectrometry to further analyze the present proteins (Lum et al. 2002; Colzani et al. 2016; Yeh et al. 2006). However, the extraction of high quality proteins for analysis faces the same challenges as genetic analysis, loss of analyzable material due to commercial processing techniques (Yeh et al. 2006; Dupree et al. 2020). Thus while methods relying on the genetic or protein profiles of ginseng have shown success, their application as a screening tool to identify ginseng species is limited by sample quality and lengthy duration of analysis.

Methods profiling a larger spectrum of ginseng’s chemical content were developed to overcome the discussed limitations of DNA and protein approaches in another field known as chemotyping. A class of secondary metabolites termed ginsenosides are assumed to be the pharmaceutically active compounds in ginseng roots and have been investigated for various biological action (Khan et al. 2015; Leung and Wong 2010). Thus, considering the unique therapeutic effects of the 3 commercially relevant ginseng species, it seems natural that each species would exhibit a distinctive profile of ginsenoside content and more broadly a unique chemical profile (Khan et al. 2015; Zhang et al. 2020). Analytically, methods including spectroscopy and mass spectrometry are commonly employed for exploring these chemical profiles. Spectroscopic methods, across a range of electromagnetic radiation from ultra-violet (UV) to terahertz (THz), have been applied in species differentiation (Chen et al. 2020; Huang 2016; Kou et al. 2021). Among these methods, Fourier-transform infrared spectroscopy (FTIR), Fourier-transform Raman spectroscopy (FT-Raman), and THz spectroscopy exhibit promise in ginseng species identification (Chen et al. 2020; Huang 2016; Kou et al. 2021). While these methods have some ability to differentiate the commercially relevant ginseng species, they provide nonspecific information pertaining to the chemical content of a sample as the profile is produced by the molecular absorption activity of all present compounds and can be complicated by poor signal resolution (Chen et al. 2020; Chen and Luthria 2013). Mass spectrometry based methods improve upon the non-specificity of spectroscopic methods by providing specific information of the mass to charge ratio (*m*/*z*) of present compound ions (Garg and Zubair 2024). There are several established mass spectrometry based methods in ginseng species and provenance identification, namely gas chromatography mass spectrometry (GC–MS), liquid chromatography mass spectrometry (LC–MS), and isotope ratio mass spectrometry among others (Huang et al. 2017; Li et al. 2000; Kim et al. 2022; Liu et al. 2022; Wang et al. 2024; Shuai et al. 2023; Chen and Bontempo 2025). While these methods are effective in differentiating the commercially relevant species, they are limited as a rapid forensic screening tool because of sample preparation, instrument run, and data processing time (Garg and Zubair 2024). In the case of GC–MS, extensive sample preparation including derivatization of ginsenosides to increase their volatility is necessary (Yu et al. 2012; Liu et al. 2016). With an estimated 2.67 million kg exported from in Canada in 2009 alone (Canada E. and C. C. 2024b), methods capable of providing a fast screening tool for ginseng species identification are essential in supporting enforcement activities.

Direct analysis in real time-time of flight mass spectrometry (DART-ToF MS) is a fast alternative to other mass spectrometry based methods with demonstrated ability in differentiating CITES listed species (Liu et al. 2016). This method is favorable due to its simple sample preparation and run times as short as 10 s per sample (McClure et al. 2015). Plant materials, such as root or wood samples, are prepared by cutting into thin slivers before holding them in a stream of excited helium gas for ionization. The generated ions were subsequently analyzed by time of flight mass spectrometery (Shuai et al. 2023; Chen and Bontempo 2025). This method has been successful in identifying other CITES listed species such as the highly valuable *Dalbergia* timbers (McClure et al. 2015; Kim et al. 2023; Brunswick et al. 2021). Furthermore, DART-ToF MS was able to determine the provenance of treasured agarwood incense as wild or cultivated (Chen and Bontempo 2025), akin to the results achieved for Mahogany using higher resolution gas chromatography time-of-flight mass spectrometry (GC-QTOF) and liquid chromatography time-of-flight mass spectrometry (LC-QTOF) procedures (Espinoza et al. 2014; Kim et al. 2023; Brunswick et al. 2021). Additionally, Lui et al. (2016) showed that DART-ToF MS was capable of analyzing ginsenoside content directly from ginseng root samples. The past success of DART-ToF MS’s in identifying CITES listed species and provenance, along with its ability to detect key variable secondary metabolites in ginseng, suggests its potential as a fast diagnostic method to screen ginseng species. A recent review of wild American ginseng (*P. quinquefolius*) identification methods highlighted the successful application of positive ionization mode DART-ToF MS, including discussion on chemotyping using bioactive components such as ginsenosides (Shaw et al. 2025). To date a comprehensive DART/ToF based method to differentiate ginseng species and their wild and cultivated specimen has yet to be published, likely due to the increasing rarity of wild American ginseng and abundant mislabeling of commercial products.

The presented study aimed to investigate the potential of DART-ToF MS for both ginseng species and provenance identification. While DART-ToF MS has demonstrated the ability to detect specific and relevant metabolites with relevance to ginseng species identification, this study aimed to use chemical profiles in place of relying on diagnostic compounds for identification due to the wide range of environmental factors which can influence metabolite content and shared chemical content between ginseng species (McGraw et al. 2013; Yu et al. 2012; Goodwin and Best 2023). As such, mass spectra acquired by DART-ToF MS from commercial American, Korean, and Chinese ginseng were compared with wild American ginseng in this study. Multivariate statistical analysis of the DART-ToF MS data allowed a visual comparison by principal component analysis (PCA) and development of a two-step discriminant analysis of principal components (DAPC) classification model. The primary objective of this study was to demonstrate the potential of DART-ToF MS as a forensic tool in screening for and identifying poaching of wild American ginseng. A two-step model was developed with the first step to distinguish between Asian ginseng (including Chinese and Korean specimens) and American ginseng. This step could offer enforcement application in identifying mislabeled commercial samples. The second step of the identification process was aimed at identifying the provenance of American ginseng samples, as wild or cultivated. This step was aimed at determining if the origin of the ginseng was related to illegal harvest.

## Methods

### Reagents and sample preparation

Caffeine D-9 was purchased from CDN isotope, Pointe-Claire, Quebec. Acetonitrile and isopropanol (OmniSolv grade) were obtained from Milllipore Sigma, Oakville, Ontario. Polyethylene gycol 600 (PEG 600) was provided by Tokyo Chemical Industry, Tokyo, Japan. Kimwipe Delicate Task Wipers were supplied by Fisher Scientific, New Hampshire, United States. Seventeen ginseng samples were analyzed to be included in the classification models: 4 Korean ginseng (*P. ginseng*), 5 Chinese ginseng (*P. notoginseng*), 5 cultivated American ginseng (*P. quinquefolius*), and 3 wild American ginseng samples (*P. quinquefolius*). Four additional samples, one from each category, were analyzed as blind quality assurance (QA) samples. Further information regarding the ginseng samples is available in the Supplemental Information (SI Table S1–S2). Ginseng root samples were cut into slivers of 0.5–1 cm length by 1–3 mm width with care to include the inside of the root and exclude the barky exterior (SI Fig. S1). The cutting tools were cleaned with OmniSolv grade isopropanol and a Kimwipe between each sample. Six replicates were cut for each sample and stored in labeled 2 ¼ × 3 ½ inch brown manilla envelopes at room temperature until data acquisition. Where ginseng specimens included several pieces of ginseng root, replicates were sampled from unique pieces for the analysis.

### DART-ToF MS data acquisition

Caffeine D-9, prepared at 10 μg/mL in acetonitrile was used to confirm the system suitability of the AccuTOF-DART 4G mass spectrometer (JEOL USA, Inc. Peabody, MA, USA) immediately prior to data acquisition. Polyethylene gycol 600 (PEG 600) was employed for confirmation of mass spectral calibration at the beginning and end of each sample batch, and the results were employed to compensate for any analytical drift. The associated msAxel@LP^®^ data processing software applied corrective mass calibration to the sample mass spectra by a < 1 min collection of a calibrant PEG 600 sample.

For DART-ToF MS sample analysis, each sample replicate was separately held vertically for ~ 10 secs in the DART-ToF MS’s helium gas stream using stainless steel tweezers (SI Fig. S2, SI Fig. S3). Six replicates where analyzed for each sample, and a PEG 600 sample was run in between each sample. Typically, three or four samples could be analyzed in a single run which lasted for a maximum of 30 min. Samples were analyzed with a DART-SVP ion source (IonSense, Saugus, MA, USA) in positive ion-mode with the source heater temperature set at 350 °C and the helium flow rate was factory preset. The spectra were recorded between 50 to 1000 m/*z* at a rate of one scan per second. The ion source was stationed to allow a 2 cm gap between the insulator cap and orifice 1. Additional instrument parameters are listed in SI Table S3.

### Heat map generation

MsAxel@LP data processing software (JEOL USA, Inc., Peabody, MA, USA) was employed to collect and process the total ion current chromatograms (TICC) produced from the ginseng samples. The extracted mass spectrum was taken over 10 s corresponding to the intensity of sample signal present in the TICC. Baseline subtraction was conducted during extraction using the background signal present between each replicate (SI Fig. S4). The produced mass spectra were exported as centroided text files and imported to Mass Mountaineer™ software to build heat maps. Heat maps of the ginseng samples included in the classification model were produced to enable both a visual assessment of the mass spectrum and to select characteristic entities for the final model. Statistically significant ions from the selected ginseng samples were identified using analysis of variance (ANOVA) before their use in multivariate statistical analysis.

### Multivariate statistical analysis

**Fig. 1:**
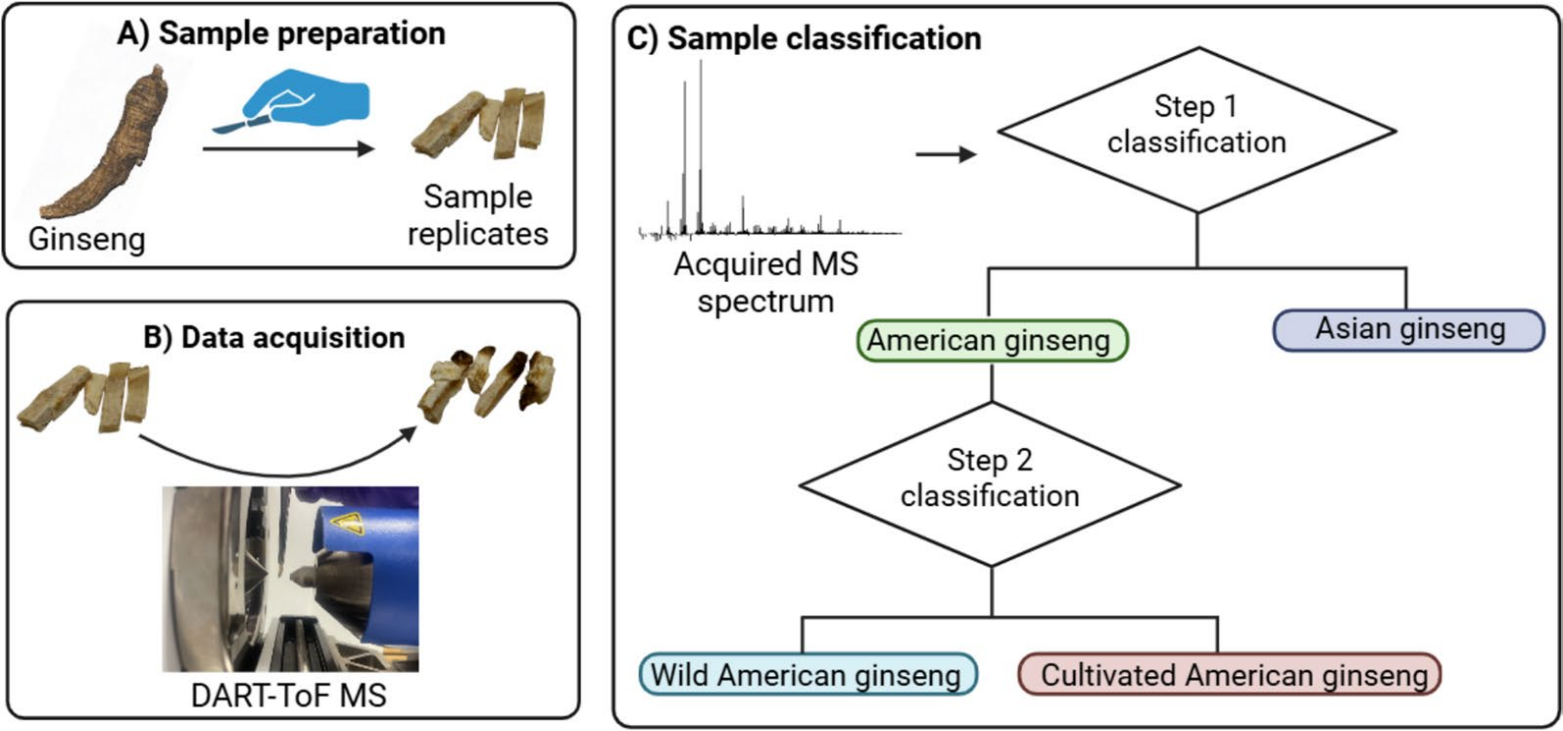
Schematic workflow using a two step classification model to identify unknown ginseng samples

Selected ions from the ginseng samples included in the classification models were used to build a two-step classification model (Fig. 1, SI Figs. S5–S6, Tables S5–S6). The initial step of the study aimed to distinguish between American ginseng (both wild and cultivated American ginseng samples) and Asian ginseng species (both Korean and Chinese ginseng samples). The secondary step was aimed at differentiation of the wild or cultivated provenance of American ginseng specimens. PCA plots were applied for each step of the model from normalized data to investigate shared characteristics within each class. The number of principal components (PC’s) was selected to cover between 75 and 100% of the variance to prevent model under and overfitting respectively (Rabinovitch et al. 2024). The generated PC’s were used to build two three-dimensional DAPC models, one for each step, with 15 mDa tolerance. Both leave one out cross validation (LOOCV) and external validation were performed to assess the accuracy and precision of DAPC models (SI Figs. S7–S10). The LOOCV was directed by the statistical analysis software program, while external validation was achieved by removing 20% of the data from the model and reintroducing it as a test set for the reproduced model.

The capability of the produced DAPC models to assign correct classes to unknown samples was investigated using QA samples which were prepared separately and unknown to the analyst. Further information regarding each classification model can be found in the supplementary information (SI Table S4).

## Results and discussion

### Mass spectra and heat map comparisons

**Fig. 2:**
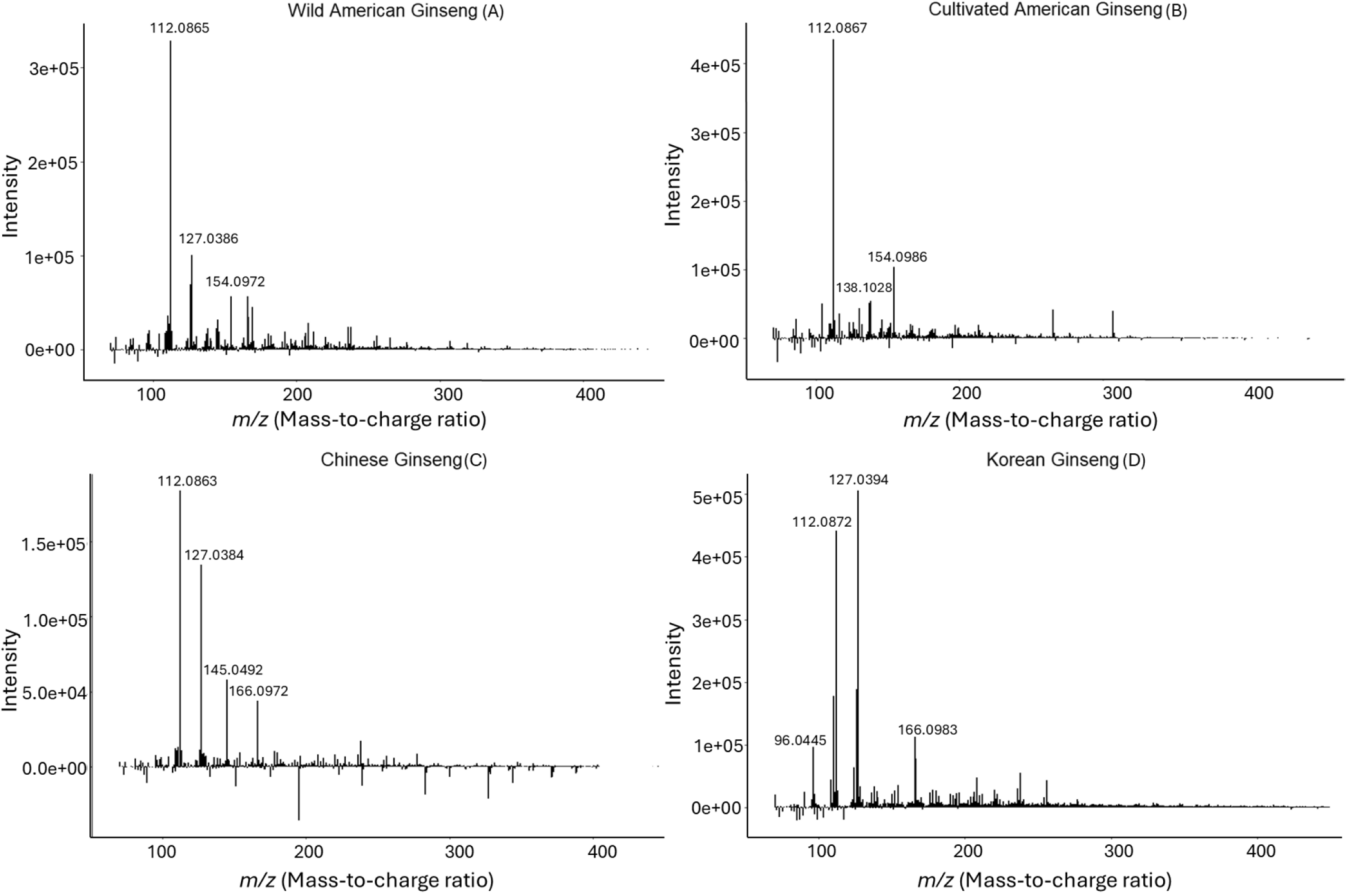
DART-ToF MS mass spectra comparison for different ginseng species and provenance including wild American ginseng (**A**), cultivated American ginseng (**B**), Chinese ginseng (**C**), and Korean ginseng (**D**)

For each step of the ginseng identification process, DART-ToF MS mass spectra from ginseng specimens were acquired. Figure 2 showcases representative mass spectra from four ginseng sample categories representative of the study, noting that there is always variability even within each species class due to source, location of sampling cut, ion response due to the characteristics of the compound and its concentration, as well as minor instrument daily variation. Nevertheless, each category or class showed a unique spectral fingerprint, which, while not immediately visually distinguishable, formed the basis of the study classification process when software algorithms were applied. Visual comparison of the American samples (Fig. 2A, B) to the Asian samples, Korean (Fig. 2D) and Chinese ginseng (Fig. 2C), allowed some observable differences in the spectra (Fig. 2A–D). For example, ion peaks 112.087 m/z and 127.039 m/z indicated general trends of relative differences in signal intensity between American and Asian sample categories (Fig. 2A–D). Ion peak 112.087 m/z generally exhibited greater relative intensity in the American specimen compared to the Asian counterpart while ion peak 127.039 m/z displayed the opposite trend (Fig. 2A–D).

The spectral difference between wild and cultivated American ginseng was more subtle as the 112.087 m/*z* ion peak elicited similar responses in both specimens (Fig. 2A, B). While 127.039 m/*z* peak displayed some variable response between the American specimens, this observed variation was not significant enough for the ion peak to be selected as a main feature between the two groups (SI Table S6). As such, these classes were more difficult to distinguish and relied on mostly in the responses of generally low abundance ions (SI Table S6). Hypothetically, this variation arose due to differences in the sample classes provenance related to location, available nutrients in the soils, local stress, or other environmental factors (Fuzzati 2004). These subtle differences observed between mass spectra of wild and cultivated American ginseng required application of the data mining component of this study, where statistical software could identify significant and distinguishable features based on the whole mass spectrums of each class.

**Fig. 3:**
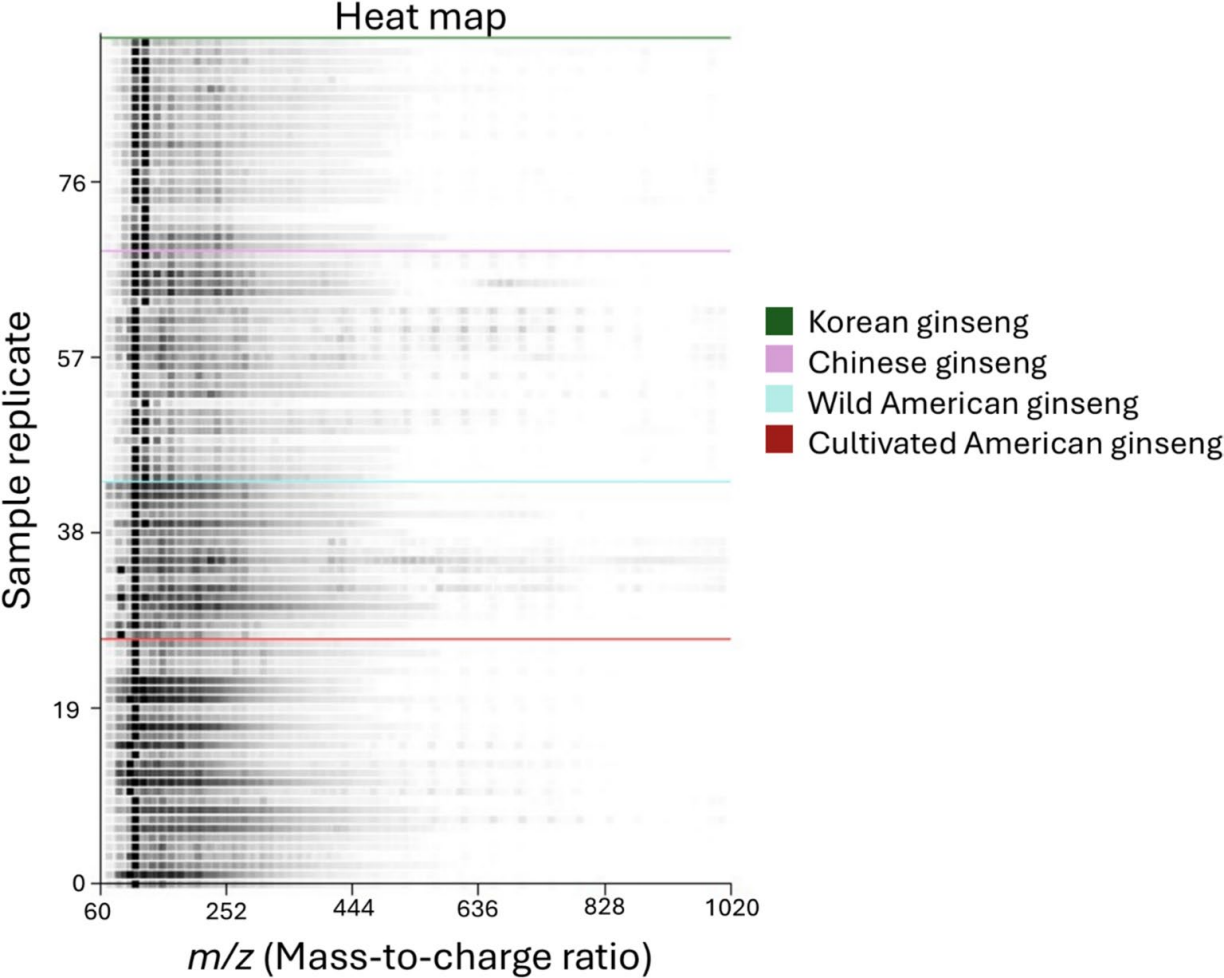
DART-ToF MS heat map of positive mass ions for all four categories of ginseng samples. Different ginseng sample categories are separated by horizontal-colored lines

Comparison of mass spectra using the Mass Mountaineer™ statistical analysis software allowed collation of the data in a variety of presentation formats and successive statistical analysis steps. Heat maps provide a preliminary visual comparison of differences in the acquired spectra (Fig. 3). The Y axis of heat map represents different sample replicates, while the X axis indicates the *m*/*z* of the detected ions, with comparative color intensity being representative of relative abundance of the ions (Rabinovitch et al. 2024). Differences in spectral pattern between the Asian ginseng (Chinese and Korean) and the American ginseng (wild and cultivated) specimens are present, but their visual distinction was not obvious (Fig. 3). In fact, only subtle differences can be observed between Chinese and Korean ginseng, and it was unclear if this was due to the supplier’s inaccuracy in reporting the provenance of each specimen. The lack of vouchered specimens available to the current study compounded this similarity issue, and a practical alternative was to pool the Chinese and Korean specimens under a single class labelled “Asian” ginseng. This would allow the study to focus on the primary goal of discerning ginseng of North American origin to develop a tool for investigating acts of illegal harvest and poaching in Canada, rather than the secondary priority of distinguishing between Chinese and Korean ginseng (Ginseng and (Panax Quinquefolius). Pdf. 2014).

Due to strong similarities in visual heat map comparisons, the study proposed differentiating ginseng based on a two-step procedure. The first step in this procedure would serve to distinguish American and Asian ginseng species in general. While this step fails to distinguish the 2 Asian species, it could still be useful in identifying mislabeled American ginseng products and aid enforcement officers in identifying illegal trade of American ginseng. Samples which are identified as American ginseng in the first step would then proceed to the second step, i.e., the specimen’s provenance identification to determine if the sample is wild or cultivated origin. While the production of heat maps was an initial step in this procedure and may not be sufficient for species identification, the characteristic mass spectral features identified within were useful for statistical model building.

### Multivariate statistics

A spectral collection range of *m*/*z* 50–1000 was used, although it was noted that most relevant ions were observed between *m*/*z* 70–500. Algorithms of the Mass Mountaineer™ software were used to conduct an unbiased selection of 2000 relevant mass ion features that enabled distinction between different ginseng sample groups. Statistical analysis by ANOVA performed on the mean abundance of each *m*/*z* between each class was used to select significantly distinct *m*/*z* with a p value of less than 0.05 for class distinction. A similar procedure was published previously in the development of identification models for other CITES listed wood species using DART-ToF MS (Espinoza et al. 2014; Kim et al. 2023).

Principal component analysis (PCA) unsupervised dimensionality reduction was used to view differences between classes in a simplified three-dimensional plot. With this process, the data was transformed into a reduced number of parameters (principal components) that accounted for the largest amount of variance. Principal components at less than 100% of variance avoided overfitting, while the *n* principal components calculated for each sample in the “training set” were used as the variables for discriminant analysis to build the models and classify unknowns. Clustering within a PCA analysis indicates the extent of shared characteristics between sample groupings, as this analysis aims to summarize both the inter and intra-population variation from large data sets without supervision (Jombart et al. 2010).

### Step 1: Classification of American and Asian ginseng

The study aimed to develop identification models under two steps; first to distinguish American and Asian specie of ginseng, and second to specifically investigate the provenance of American ginseng specimen. Reasoning for this two-step procedure was to prevent the misclassification of wild and cultivated American ginseng as their subtle mass spectral differences required a focused statistical analysis for clarity.

**Fig. 4:**
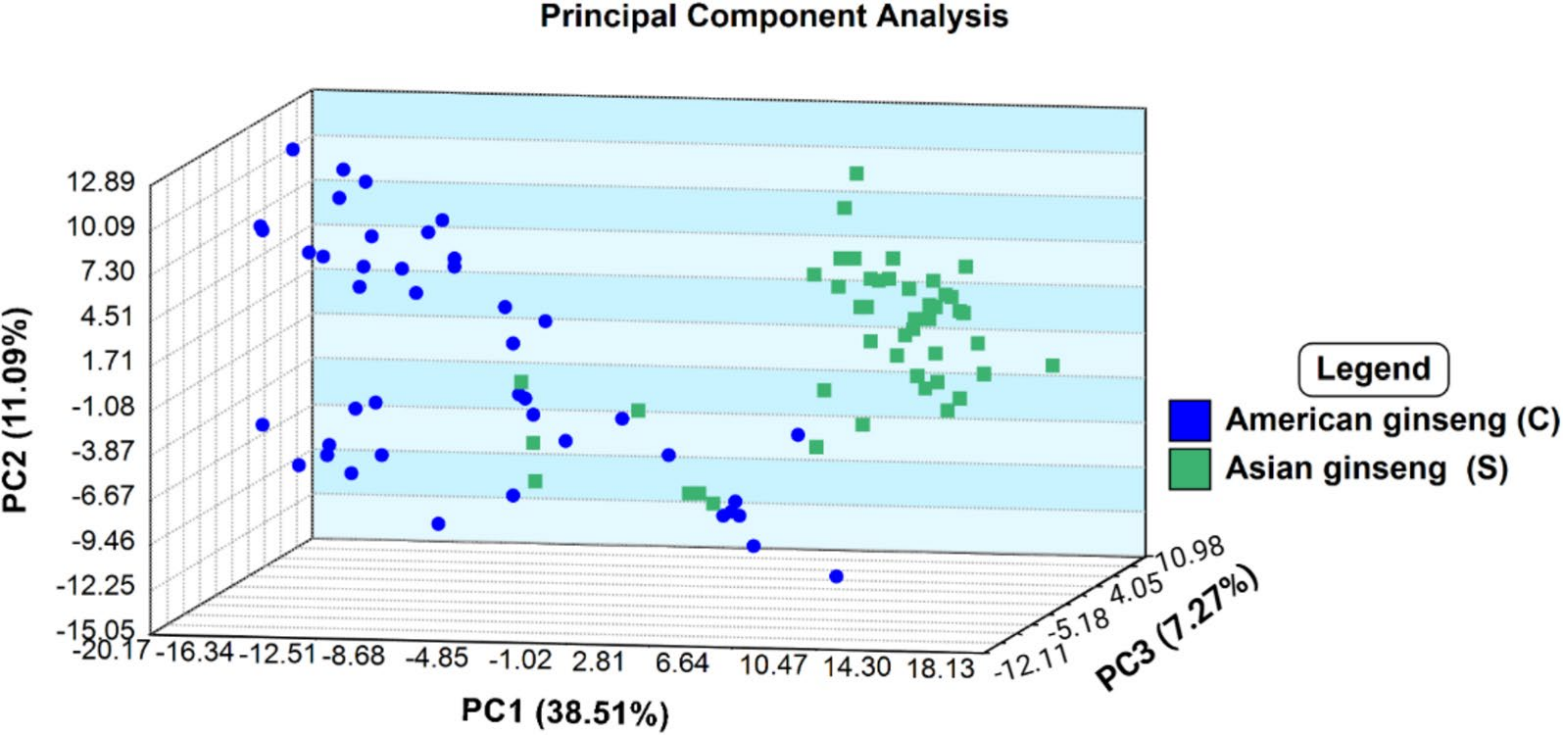
PCA analysis showing variability in the Asian (Korean and Chinese) and American ginseng groupings

**Fig. 5:**
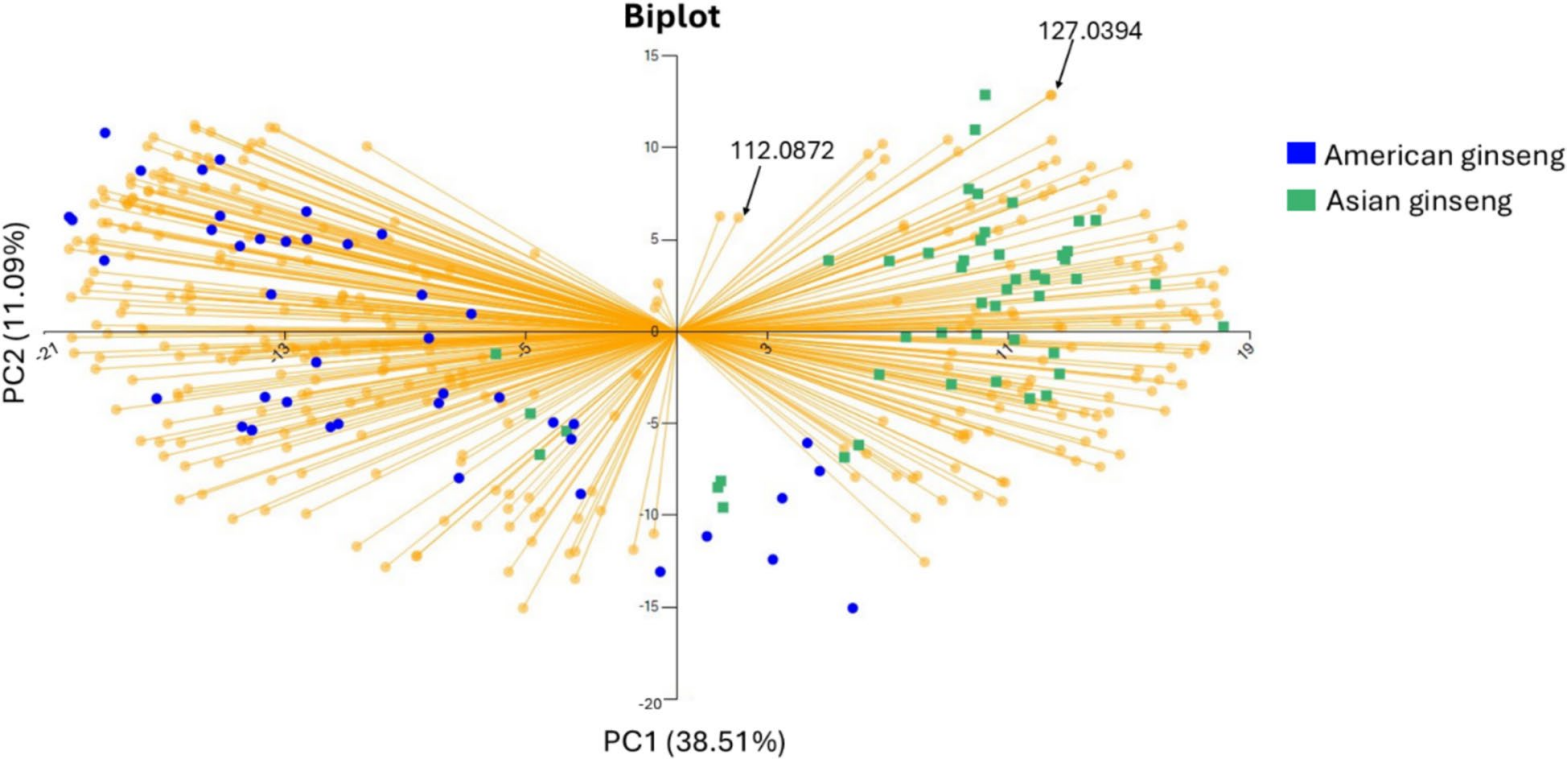
PCA loading plot for classification of American and Asian (Korean and Chinese) ginseng groupings

Observation of the clustering and variability within the American and Asian sample groupings was apparent in the PCA analysis plot (Fig. 4). The inclusion of both Chinese and Korean ginseng under one category as “Asian” ginseng facilitated the separation of cluster groupings (Fig. 4). The clustering observed in Fig. 4 highlights the tendency of American and Asian species to generally cluster with their respective class, however, some overlap between the classes is apparent. The overlap is likely due to the shared high relative intensity of ion peaks at *m*/*z* 112.087 across all ginseng sample types (Fig. 1A–D). Additionally, the slightly elevated relative intensity of ion peaks at 127.039 m/*z* in the wild American ginseng is likely less significant and is largely absent from most cultivated American ginseng specimen spectra’s (Fig. 1b). These peaks, despite their elevated response across classes, were found to be distinguishing features between American and Asian ginseng specimens (Fig. 1, SI Table S5) but excluded by the software for distinguishing wild and cultivated American ginseng (discussed in next section; Table S6). The loading plot represented in Fig. 5 indicates that these mass ions are correlated and positively contribute to both PC1 and PC2 due to their shared position in the top right quadrant (Fig. 5). As such, it is possible that due to their presence in all ginseng sample types, these ions may contribute to the overlap of some replicates in the produced PCA plot (Figs. 4, 5). Additionally, American ginseng samples in the PCA are observed in a greater dispersion than the Asian ginseng class, likely due to the inclusion of both cultivated and wild American ginseng samples in this class (Fig. 4). Past studies investigating the provenance of wild and cultivated CITES listed tree species with DART-ToF MS and multivariate analysis have observed the tendency for wild populations to demonstrate greater variation in acquired mass spectra and dispersion in multivariate statistical models than cultivated samples (Espinoza et al. 2014; Kim et al. 2023). This is likely a consequence of the greater variation in environmental conditions which wild American ginseng specimens may have experienced in comparison to that of cultivated specimen grown in largely controlled environments contributing to the chemical profiles of the specimen (Fuzzati 2004). Note that the current method may be applicable for wild Korean ginseng when sufficient specimens become available as its Russian Federations population is also listed under CITES Appendix II (Panax ginseng 2024).

**Fig. 6:**
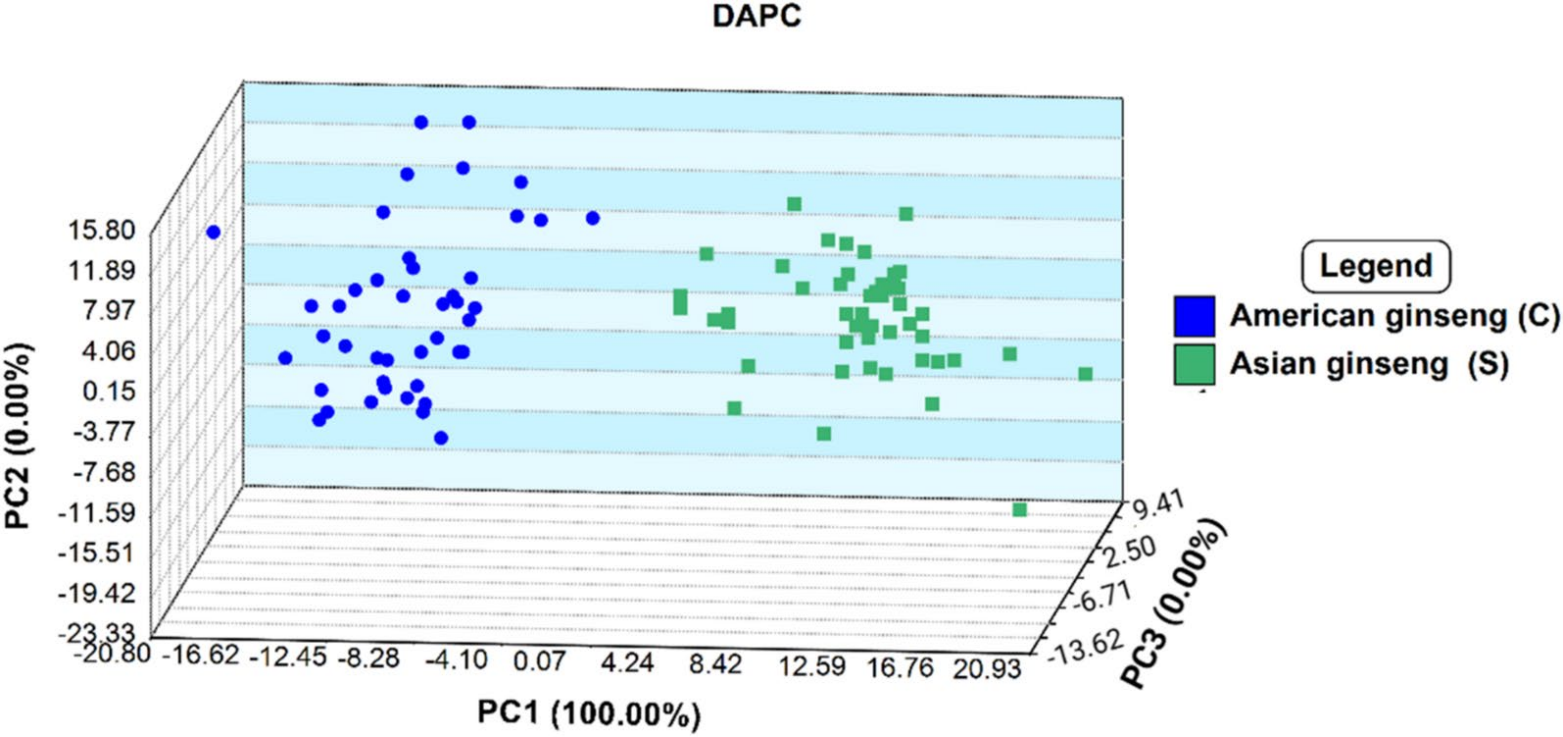
Three-dimensional scatter plot for DAPC analysis for stage 1 of the classification model to differentiate between Asian (Korean and Chinese) and American ginseng

Information yielded from the initial PCA analysis was further analyzed to determine the best approach for identification modelling. Of the various modelling processes available, Discriminant Analysis of Principal Components (DAPC) offered the best supervised description of clusters. From the data set, 17 PC’s covering 85.33% of the selected features variance were employed to produce DAPC three-dimensional plots (Fig. 6). The increased ability of DAPC methods to categorize data allowed for greater distinction between clusters of Asian and American ginseng species than that observed in PCA analysis.

The step 1 DAPC model was validated using both LOOCV, where each data point was removed individually before being classified as an unknown sample, and external validation, in which 20% of the data from the model was removed and reintroduced as unknown samples. With a total of 18 samples included in the step 1 model, LOOCV scored 96.7% (SI Table S4, SI Fig. S8). Of the LOOCV data, 2 of 5 Chinese ginseng and 1 of 3 wild American were misassigned as American and Asian ginseng respectively (SI Fig. S8). By comparison, external validation yielded an overall score of 88.9% (SI Table S4), indicating the model’s ability to classify unknown samples as Asian or American ginseng.

Confidence in the model was further investigated by analysis of four quality assurance (QA) samples whose identities were blind to the primary analyst (SI Table S2). The QA samples from the four different species categories were introduced to the step 1 model as unknown samples for classification (Table 1, SI Fig. S11, SI Table S7). Each sample type was analyzed using 6 replicates for repeatability. The rate of agreement was calculated by dividing the number of correct assignments by the total number of replicates. One replicate in QA1 (cultivated American) and QA4 (Chinese) sample groupings were misidentified, resulting in the lower percentage agreements among replicates at 83% rather than 100% (Table 1, SI Table S7). These misidentifications may be due the minimal sample overlap first observed in the step 1 PCA plot which suggested that the American and Asian species shared some characteristics (Fig. 4). Overall, step 1 was able to successfully identify all four QA samples with the lowest rate of agreement for the correct identification among sample replicates being 83.3% (Table 1). The ability of step 1 to correctly identify blind QA samples further demonstrated the model’s capability to distinguish between Asian and American ginseng species.

**Table 1:** Identification of blind QA samples from all species and provenance used for validation of the Step 1 DAPC model to determine Asian or American origin

QA sample identity	Sample name	Sample identity	Percent of sample replicates correctly identified (%)
1	QUI_C_05	Cultivated American ginseng (*P. quinquefolius*)	83
2	GIN_C_03	Korean ginseng (*P. ginseng*)	100
3	QUI_W_02	Wild American ginseng (*P. quinquefolius*)	100
4	NOT-C-02	Chinese ginseng (*P. notoginseng*)	83

### Step 2: Classification of wild and cultivated American ginseng

**Fig. 7:**
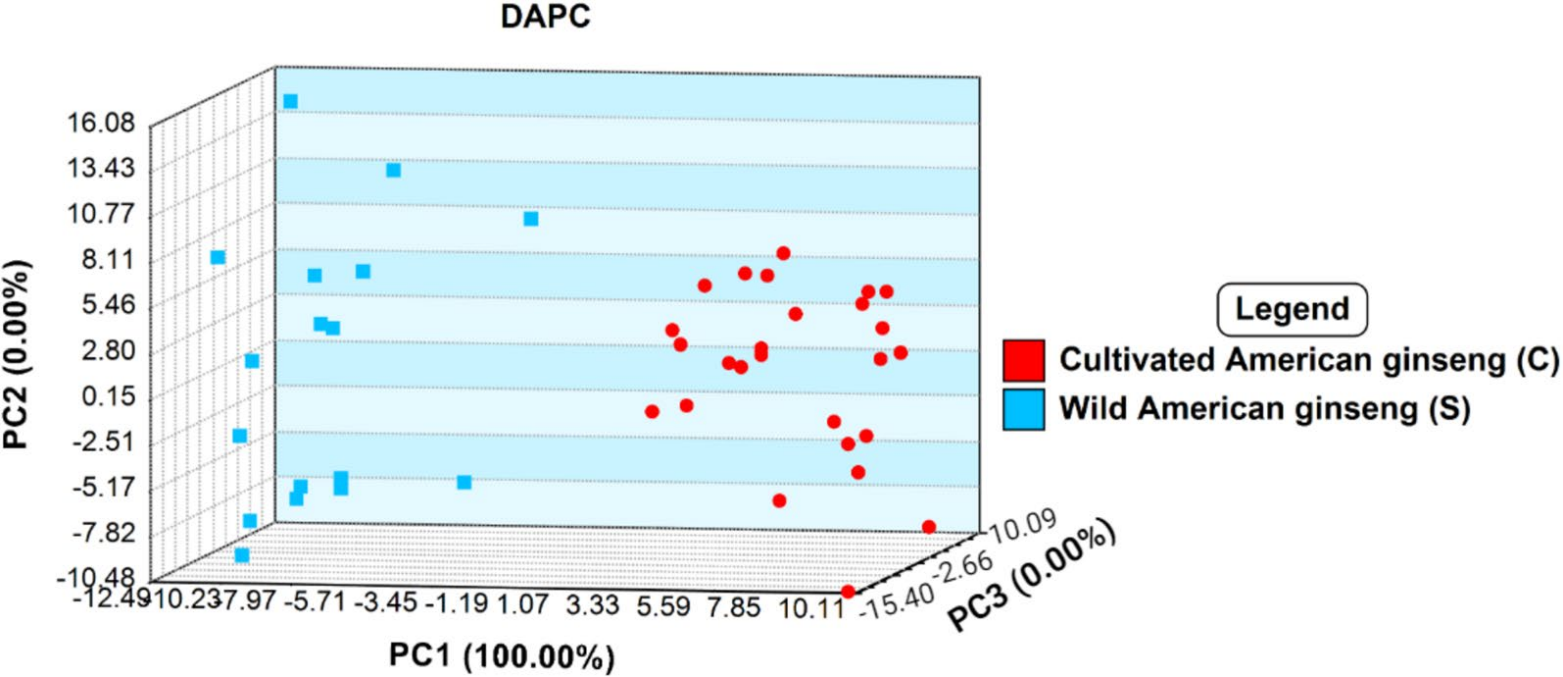
PCA analysis showing the variability in wild and cultivated American ginseng

Following identification of American from Asian ginseng, step 2 of the current study was aimed at distinguishing between wild and cultivated American ginseng specimens. Past studies employing DART-ToF MS for provenance identification have demonstrated that wild populations generally exhibit greater spectral variability and dispersion in multivariate statistical models compared to cultivated samples of the same species (Espinoza et al. 2014; Kim et al. 2023). This is likely a consequence of the wide variation in environmental conditions under which wild flora grow in comparison to cultivated flora under controlled grown conditions (Fuzzati 2004). In the current study, the step 2 comparison of wild and cultivated American ginseng by PCA supported this view (Fig. 7). In fact, clustering of each grouping suggested the potential for these populations to be distinguished despite some overlap between the populations (Fig. 7).

**Fig. 8:**
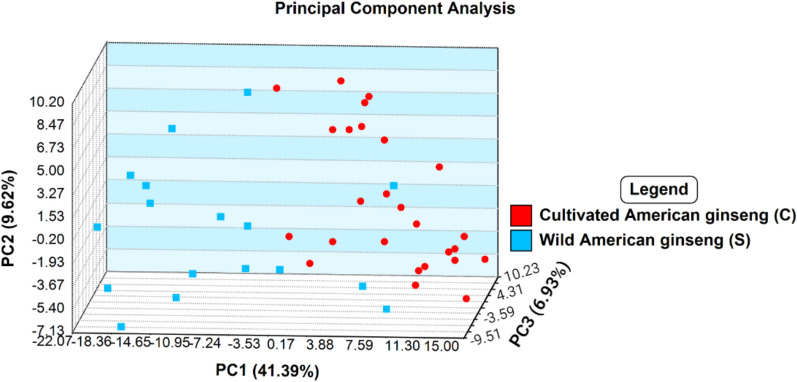
Three-dimensional scatter plot for DAPC analysis for step 2 of the classification model to differentiate between wild and cultivated American ginseng

While the PCA plots were able to summarize the differences between classes for both steps of the model, this type of analysis is unsupervised and is primarily useful for exploring trends in large data sets rather than building classification models (Jombart et al. 2010). DAPC analysis, building upon the PC’s, was employed to build the final classification models. Step 2 data produced 13 PC’s covering 85.59% of the variance which was used to build the three-dimensional DAPC model (Fig. 8). Clearly, the use of DAPC demonstrated superior clustering of wild or cultivated American ginseng classes compared to the PCA plot.

Step 2 model validation scored 86.4% in the LOOCV (SI Table S4, SI Figure S10) and 77.8% in the external validation (SI Table S4, SI Fig. S9). These reduced scores in comparison to the step 1 model validation, at 96.74% and 88.98% LOOCV and external validation respectively, were inferred to be due to the increased similarity in abundant mass spectral features between wild and cultivated American ginseng (SI Table S4). Overall, these results highlighted the model’s ability to identify wild and cultivated samples with high accuracy. Successful classification of American ginseng specimen as wild or cultivated is a significant advance in combating illegal harvesting and protecting the remaining wild ginseng populations in Canada.

For confirmation of the applicability of the step 2 model, data was acquired from four blind QA samples introduced to the step 1 model as unknown samples for classification (Table 1, Fig. S11, Table S5). Step 1 was able to identify all four QA samples with the lowest rate of agreement for the correct identification among sample replicates being 83.33% (Table 1). Both QA1 and QA3 samples were identified as American ginseng under Step 1 classification model (Table 1). Consequentially, these samples were progressed to step 2 to be classified as either wild or cultivated American ginseng. The step 2 model was able to correctly assign both QA1 and QA3’s identities as cultivated or wild respectively with 100% agreement among both sample’s replicates (Table 2, SI Fig. S12, SI Table 8). These results supported the view that DART-ToF MS is sensitive enough to determine both ginseng species and provenance of unknown samples.

**Table 2:** QA sample identification results for Step 2 analysis

QA sample identity	Sample name	Sample identity	Percent of sample replicates correctly identified (%)
1	QUI_C_05	Cultivated American ginseng (*P. quinquefolius*)	100
3	QUI_W_02	Wild American ginseng (*P. quinquefolius*)	100

### Application of DART-ToF MS in conservation

The potential of DART-ToF MS as a forensic tool is quickly becoming recognized with application in the identification of endangered and CITES listed species (Kim et al. 2023). Past studies have successfully demonstrated the ability of DART-ToF MS to distinguish between species and provenance of other CITES listed species including *Dalbergia* timbers, agarwood, mahogany, and corals (McClure et al. 2015; Espinoza et al. 2014, 2015; Kim et al. 2023; Brunswick et al. 2021; Hayes et al. 2021). The success of the developed two-step model for accurately identifying endangered wild American species further expands the application of DART-ToF MS as a forensic tool to protect CITES listed species. From a conservation perspective, the success of these statistical models may increase the capability of enforcement officers to identify and prosecute poaching of wild American ginseng and discourage future illegal harvesting. The fast sample preparation, instrument run, and data analysis by DART-ToF MS highlights the method’s suitability as an efficient forensic screening tool. While the assignment of the QA samples identities in this study was successful, future improvements to this model to expand the amount of ginseng specimens and incorporate vouchered specimens is required to increase confidence in the model’s identity assignments. It is recognized that DART-ToF MS does not necessarily compete with the superior sensitivity and precision of other GC or LC based mass spectrometers but is valuable in its rapidity as a screening tool complementary to other instrumentation. A combination of techniques offers readily defensible evidence to support the legal infrastructure protecting wild American ginseng in Canada. The use of complimentary supporting techniques is often used in other areas of forensics, such as oil spill where the international standard being the European Committee for Standardization (CEN) method includes both GC-FID and GC/MS (CEN 2021; Chua et al. 2022). The coupling of DART-TOFMS as a screening tool together with a robust investigation by GC–MS to identify and provide evidence of poaching events in Canada may deter such illegal activities in the future.

## Conclusion

Legislation protecting endangered and CITES listed species such as wild American ginseng requires support from all available tools and methods of forensic investigation. This study aimed to determine if DART-ToF MS can be applied as a rapid screening tool for differentiating ginseng species and identifying poached wild American ginseng. Ultimately, mass spectra acquired by DART-ToF MS from ginseng specimen were used to build two models differentiating between Asian and American ginseng first before distinguishing wild and cultivated American ginseng by their chemical fingerprints. The developed models were validated by leave-one-out and external validation and were further successfully applied to classifying blind unknown QA samples. The potential of DART-ToF MS as a forensic screening tool for identifying the poaching of wild American ginseng was demonstrated. To develop this tool for real world use, the data set would benefit from expansion to include more vouchered specimens to improve method robustness, accuracy, and applicability to common ginseng root samples with variable ages, moisture levels, and storage conditions. Ultimately, DART ToF–MS shows great potential as a powerful forensic tool available to enforcement officers, analytical chemists, and conservationists in combating poaching and illegal trade of CITES listed species such as wild American ginseng**.**

## Supplementary Information

Additional file 1.

## Data Availability

Further data is available in the Supplemental Information or upon request from the author(s).

## Abbreviations

TM: Traditional medicines
CITES: Convention on International Trade in Endangered Species of Wild Fauna and Flora
WAPPRIITA: Wild Animal and Plant Protection and Regulation of International and Interprovincial Trade Act
AFLP: Amplified fragment-length polymorphism
RAPD: Random amplified polymorphic DNA
PCR: Polymerase chain reaction
DNA: Deoxynucleic acid
SDS-PAGE: Sodium dodecyl sulfate–polyacrylamide gel electrophoresis
2DE: 2-Dimensional gel electrophoresis
UV: Ultraviolet
THz: Terahertz
FTIR: Fourier-transform infrared spectroscopy
FT-Raman: Fourier-transform Raman spectroscopy
*m*/*z*: Mass-to-charge ratio
GC–MS: Gas chromatography mass spectrometry
LC-MS^2^: Liquid chromatography tandem mass spectrometry
HPLC ToF–MS: High performance liquid chromatography time of flight mass spectrometry
DART-ToF MS: Direct analysis in real time-time of flight mass spectrometry
GC-QTOF: Gas chromatography time-of-flight mass spectrometry
LC-QTOF: Liquid chromatography time-of-flight mass spectrometry
PCA: Principal component analysis
DAPC: Discriminant analysis of principal components
PEG 600: Polyethylene glycol 600
QA: Quality assurance (unknown sample prepared to validate the method)
TICC: Total ion current chromatogram
ANOVA: Analysis of variance
PC: Principal component
LOOCV: Leave one out cross validation
