# Supplementary material for: A high throughput ambient mass spectrometric approach for identifying the poaching of wild american ginseng
Source: Environ Syst Res (Heidelb). 2025 Dec 20;14(1):31. doi: 10.1186/s40068-025-00414-6 (PMC12718241; doi:10.1186/s40068-025-00414-6)

**A High Throughput Ambient Mass Spectrometric Approach for Identifying the Poaching of Wild American Ginseng**

Julia E. Shaw^a^, Pamela Brunswick^b^, Jolene Lesuk^b^, Lola Rabinovitch^b^, Taylor Filewood^b^, Honoria Kwok^b^, Jeffrey Yan^b^, Robert Cody^c^, Tao Huan^a^*, Dayue Shang^b^*

^a^ Department of Chemistry, Faculty of Science, University of British Columbia, 2036 Main Mall, Vancouver, BC, Canada, V6T 1Z1

^b^ Science and Technology Branch, Pacific Environmental Science Centre, Environment and Climate Change Canada, Pacific and Yukon Laboratory for Environmental Testing, North Vancouver, BC, Canada, V7H 1V2

^c^JEOL USA Inc, Peabody, Massachusetts, United States of America

*Corresponding authors Tao Huan [thuan@chem.ubc.ca](mailto:thuan@chem.ubc.ca) and Dayue Shang [Dayue.Shang@ec.gc.ca](mailto:Dayue.Shang@ec.gc.ca)

**Supplemental Information:**

**Table S1:** Ginseng sample information used to construct both step 1 (species) and step 2 (provenance) classification models.

| **Sample** | **Species** | **Step 1** | **Step 2** | **Source** | **Harvest Location** |
| --- | --- | --- | --- | --- | --- |
| GIN_C_01 | Korean ginseng (*P. ginseng*) | Asian ginseng | Not included | Amazon.ca | Korea |
| GIN_C_02 | Korean ginseng (*P. ginseng*) | Asian ginseng | Not included | Amazon.ca | Korea |
| GIN_C_04 | Korean ginseng(*P. ginseng*) | Asian ginseng | Not included | Ka Wing Hong US | Korea |
| GIN_C_05 | Korean ginseng (*P. ginseng*) | Asian ginseng | Not included | Amazon.ca | Korea |
| NOT_C_01 | Chinese ginseng (*P. notoginseng*) | Asian ginseng | Not included | Amazon.ca | China |
| NOT_C_03 | Chinese ginseng (*P. notoginseng*) | Asian ginseng | Not included | Herbal Care, Jingling | China |
| NOT_C_04 | Chinese ginseng (*P. notoginseng*) | Asian ginseng | Not included | Herbal Care, Jingling | China |
| NOT_C_05 | Chinese ginseng (*P. notoginseng*) | Asian ginseng | Not included | Ulike | China |
| NOT_C_06 | Chinese ginseng (*P. notoginseng*) | Asian ginseng | Not included | MELITT | China |
| QUI_C_01 | Cultivated American ginseng (*P. quinquefolius*) | American ginseng | Cultivated American ginseng | Dairy Land Management LLC | Wisconsin, USA |
| QUI_C_02 | Cultivated American ginseng (*P. quinquefolius*) | American ginseng | Cultivated American ginseng | Chinapoarl | USA |
| QUI_C_03 | Cultivated American ginseng (*P. quinquefolius*) | American ginseng | Cultivated American ginseng | Amazon.ca | USA |
| QUI_C_06 | Cultivated American ginseng (*P. quinquefolius*) | American ginseng | Cultivated American ginseng | Amazon.ca | USA |
| QUI_C_11 | Cultivated American ginseng (*P. quinquefolius*) | American ginseng | Cultivated American ginseng | Herbsmate, | USA |
| QUI_W_01 | Wild American ginseng (*P. quinquefolius*) | American ginseng | Wild American ginseng | Donation by Planentiuim Meise | Quebec, Canada |
| QUI_W_03-29 | Wild American ginseng (*P. quinquefolius*) | American ginseng | Wild American ginseng | Donation by Jean-François Dubois, Canadian Enforcement | Canada (Ontario or Quebec). |
| QUI_W_30 | Wild American ginseng (*P. quinquefolius*) | American ginseng | Wild American ginseng | N. Cotter, Alberta Wildlife enforcement | Canada |

**Table S2:** Ginseng sample information used as unknown QA samples to be classified by the produced models.

| **Sample Name** | **Species** | **QA Number** | **Source** | **Harvest Location** |
| --- | --- | --- | --- | --- |
| GIN_C_03 | Korean ginseng (*P. ginseng*) | 2 | Amazon.ca | Korea |
| NOT_C_02 | Chinese ginseng (*P. notoginseng*) | 4 | Herbal Care, Jinling | China |
| QUI_C_05 | Cultivated American ginseng (*P. quinquefolius*) | 1 | Vigorwell | Ontario, Canada |
| QUI_W_02 | Wild American ginseng (*P. quinquefolius*) | 3 | Donation from Planentiuim Meise | Missouri, USA |

**
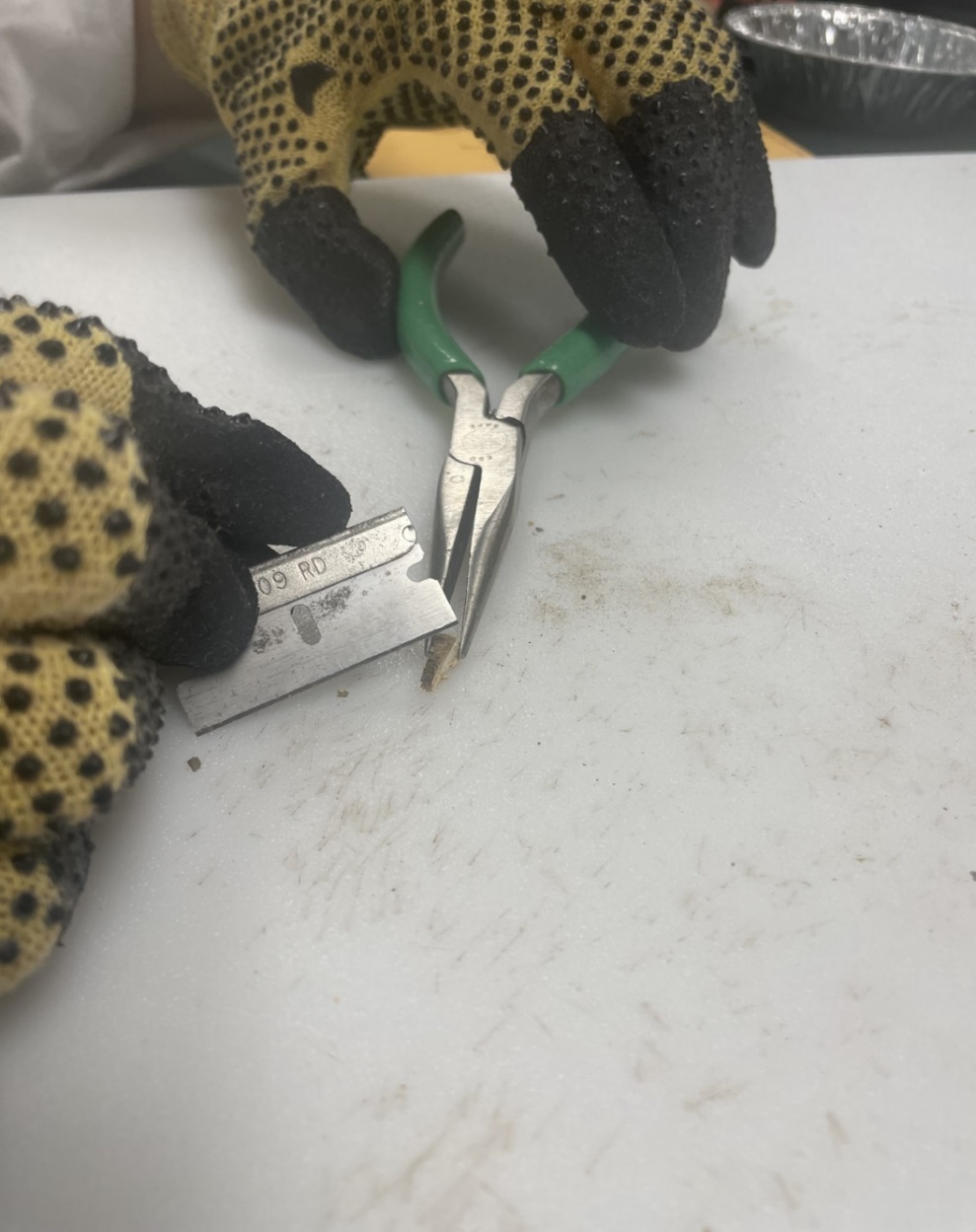
Figure S1:** Image of ginseng sample cutting technique.


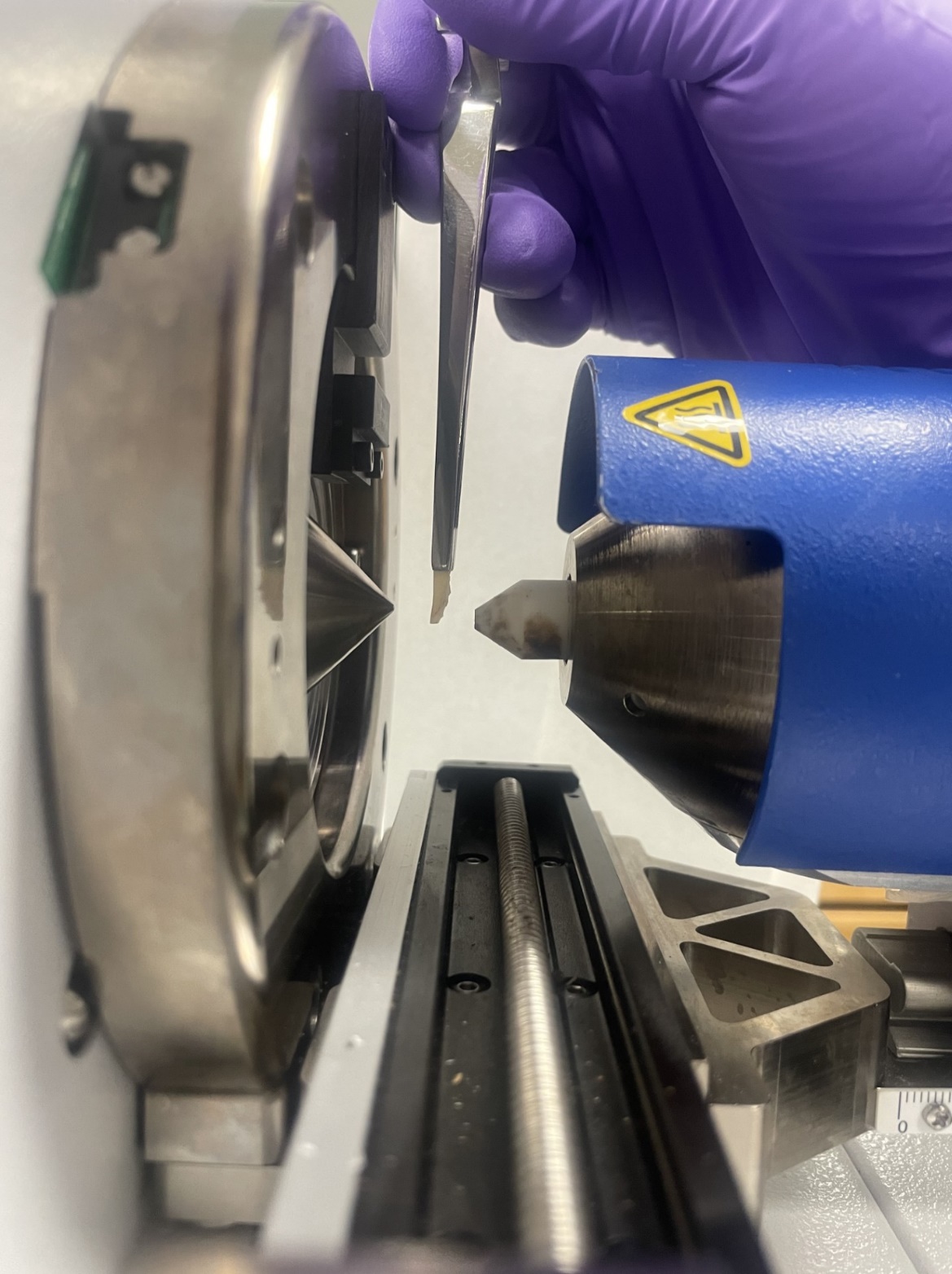


**Figure S2:** Position of ginseng sample held in DART-ToF MS gas stream.


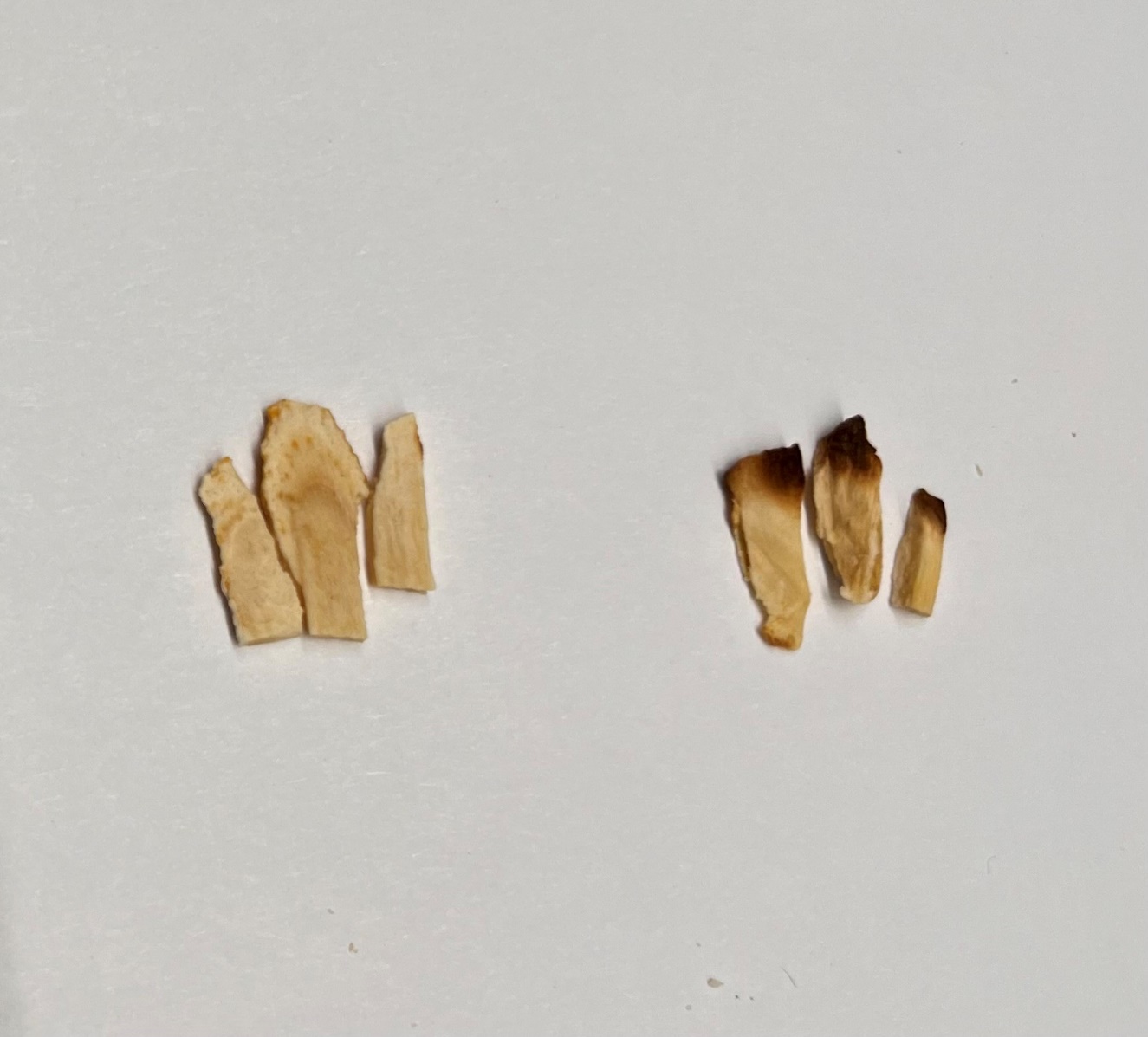


**Figure S3:** Example of ginseng sample replicated before and after data acquisition by DART-ToFMS.

**Table S3:** AccuTOF-DART 4G mass spectrometer operating parameters.


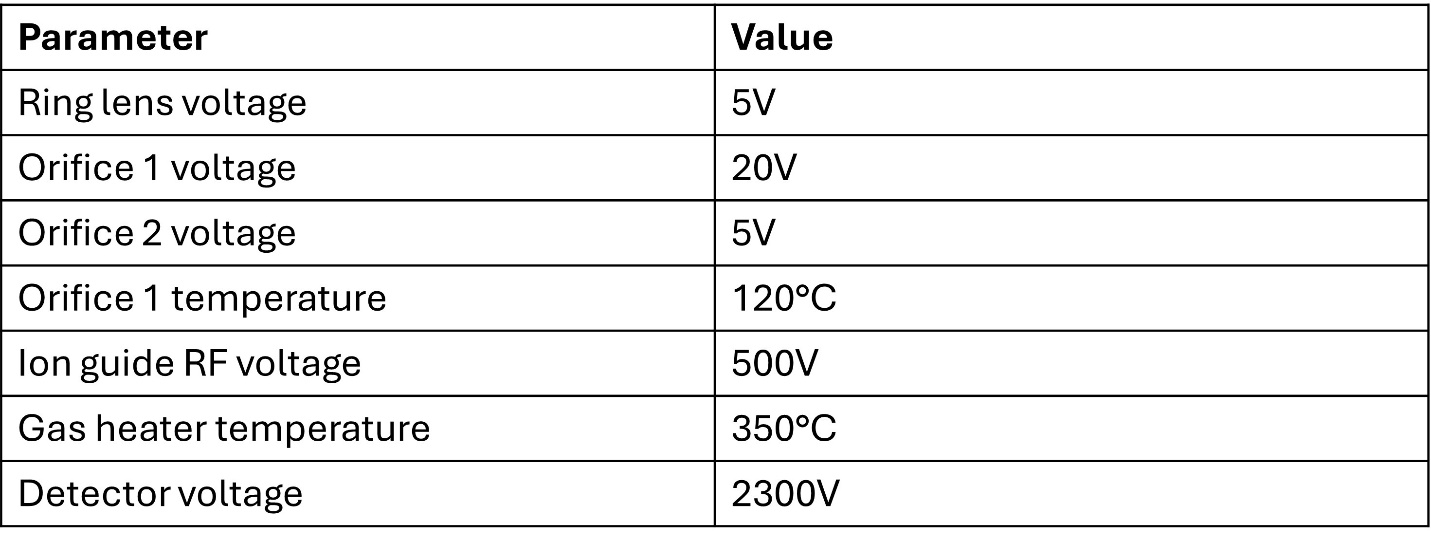


**Table S4:** Ions and parameters of the Discriminant Analysis of Principal Components (DAPC) step 1 and step 2 classification models.

| **Model** | **Number of features** | ***m/z* range** | **Number of files** | **Number of Principle Components (PC)** | **Variance covered by PC’s (%)** | **LOOCV score (%)** | **External validation score** |
| --- | --- | --- | --- | --- | --- | --- | --- |
| Step 1- Species | 323 | 72.08-570.36 | 94 | 17 | 85.53 | 96.74 | 88.89 |
| Step 2- Provenance | 369 | 81.03-575.50 | 44 | 13 | 85.59 | 86.36 | 77.78 |

**
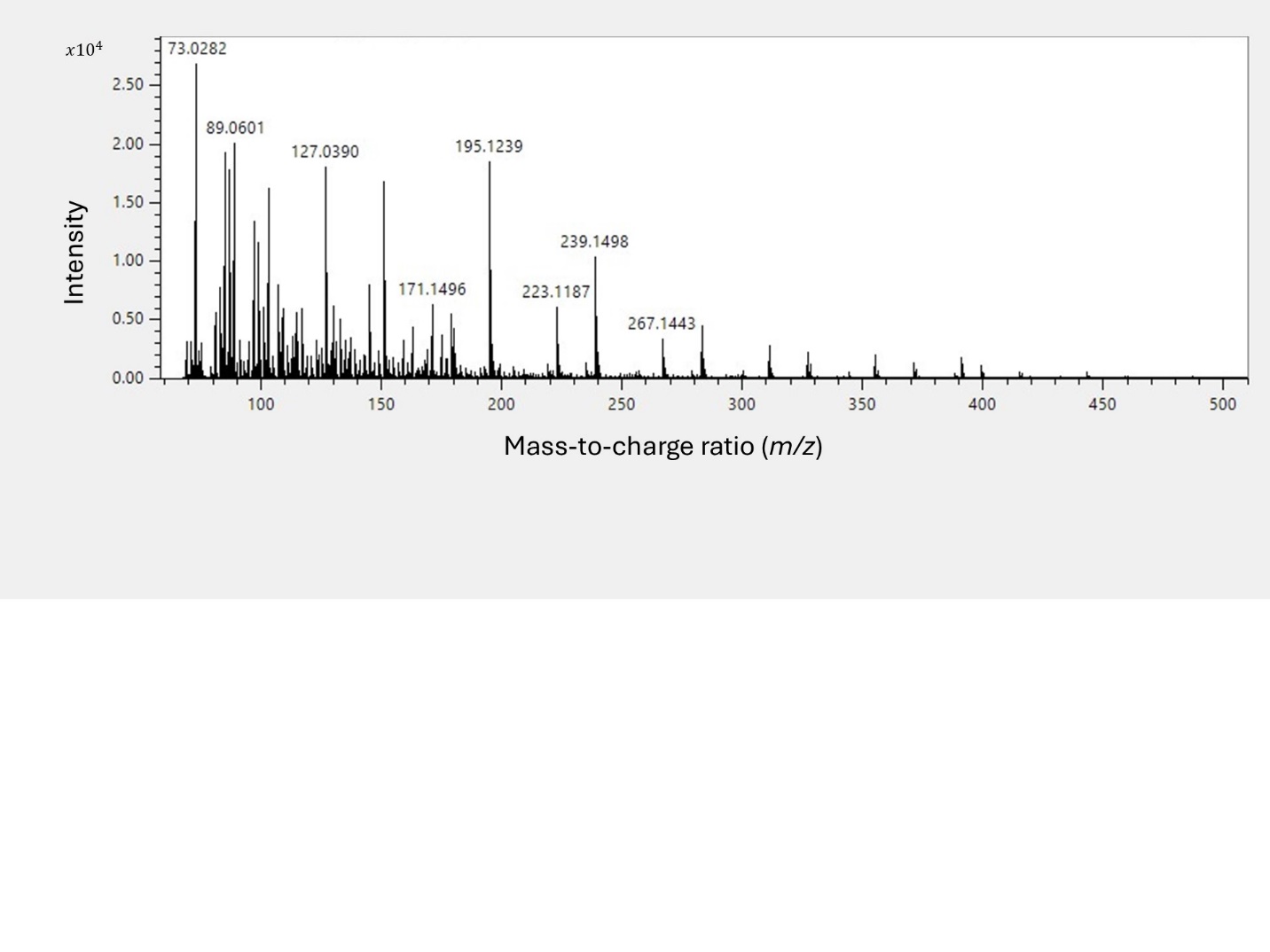
**

**Figure S4:** Example mass spectrum of background noise used for baseline subtraction. Background spectra was subtracted from all sample spectra.


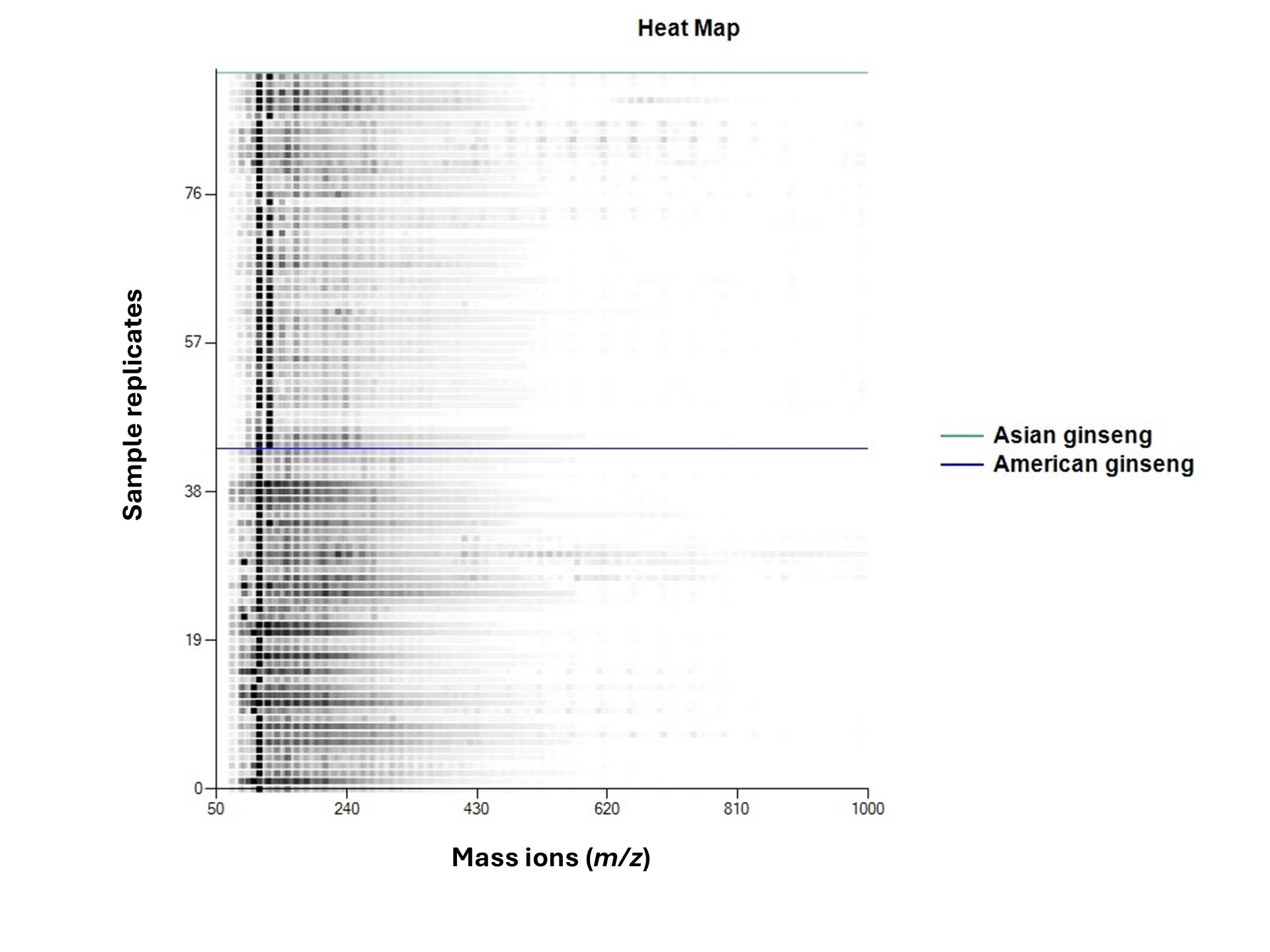
**Figure S5**: Heat map of mass ions observed for step 1 (species) classification model.

**Table S5:** List of selected ions used to build step 1 PCA and DAPC. The fold values refer to the difference in mean abundance for that m/z between the two classes and the p value from analysis by ANOVA.

| **Selected m/z's** | **P value** | **Fold** |
| --- | --- | --- |
| 72.08041 | 0 | 8.9 |
| 74.05989 | 0.04 | 1.46 |
| 81.03378 | 0 | 6.14 |
| 84.04453 | 0 | 4.24 |
| 84.08086 | 0 | 2.51 |
| 86.06039 | 0 | 2.77 |
| 86.09673 | 0 | 2.56 |
| 87.04432 | 0 | 4.22 |
| 89.10751 | 0 | 66.79 |
| 90.05489 | 0 | 3.38 |
| 90.09132 | 0.02 | 4.9 |
| 91.09444 | 0.02 | 6.82 |
| 95.05958 | 0 | 1.88 |
| 97.0275 | 0 | 6.13 |
| 97.07553 | 0.01 | 1.43 |
| 98.09601 | 0 | 2.38 |
| 99.04346 | 0 | 6.96 |
| 103.08659 | 0 | 20.22 |
| 104.07003 | 0 | 3.13 |
| 105.07349 | 0 | 4.52 |
| 108.04442 | 0 | 3.84 |
| 109.02815 | 0 | 10.58 |
| 109.07573 | 0 | 1.72 |
| 110.06038 | 0.01 | 1.42 |
| 110.08072 | 0 | 2.64 |
| 111.044 | 0 | 1.97 |
| 111.0917 | 0 | 2.7 |
| 112.0872 | 0.02 | 1.29 |
| 113.08874 | 0.03 | 1.25 |
| 116.07027 | 0 | 2.71 |
| 118.08598 | 0 | 1.99 |
| 120.06602 | 0 | 1.82 |
| 121.07593 | 0 | 2.35 |
| 123.05543 | 0 | 2.54 |
| 123.09142 | 0 | 2.98 |
| 124.07618 | 0 | 1.78 |
| 124.09595 | 0 | 42.38 |
| 124.15247 | 0.01 | 2.16 |
| 125.07071 | 0 | 1.61 |
| 125.10709 | 0 | 8.59 |
| 126.05534 | 0 | 3.39 |
| 126.10265 | 0 | 2.4 |
| 127.03941 | 0 | 14.07 |
| 127.07364 | 0 | 2.61 |
| 127.08885 | 0 | 2.18 |
| 128.04245 | 0 | 14.44 |
| 128.07497 | 0 | 1.84 |
| 128.10699 | 0 | 2.18 |
| 129.06378 | 0 | 2.01 |
| 129.10214 | 0 | 4.93 |
| 130.08603 | 0.02 | 1.34 |
| 130.12248 | 0 | 3.11 |
| 132.1017 | 0 | 2.13 |
| 133.07442 | 0.03 | 1.54 |
| 135.09148 | 0 | 2.27 |
| 137.10724 | 0 | 6.96 |
| 138.10294 | 0 | 2.87 |
| 139.08688 | 0 | 1.88 |
| 139.12267 | 0 | 21.79 |
| 140.07094 | 0 | 1.76 |
| 140.11804 | 0 | 1.79 |
| 141.10269 | 0 | 5.68 |
| 144.06549 | 0 | 2.03 |
| 144.10182 | 0 | 2.7 |
| 145.04944 | 0 | 3.85 |
| 146.16473 | 0 | 10 |
| 147.09012 | 0 | 1.95 |
| 148.08875 | 0 | 1.64 |
| 149.10727 | 0 | 5.75 |
| 150.09636 | 0 | 1.43 |
| 151.12337 | 0 | 26.12 |
| 152.08029 | 0 | 2.02 |
| 152.11778 | 0 | 5.45 |
| 153.10254 | 0 | 3.44 |
| 153.13852 | 0 | 16.5 |
| 154.09476 | 0 | 1.42 |
| 155.09575 | 0.01 | 1.51 |
| 155.11815 | 0 | 15.78 |
| 156.10266 | 0.01 | 1.33 |
| 158.1178 | 0 | 2.79 |
| 161.06044 | 0 | 11.19 |
| 161.10663 | 0 | 2.92 |
| 161.12856 | 0.02 | 7.31 |
| 163.12326 | 0 | 22.44 |
| 164.07303 | 0 | 6.84 |
| 164.10904 | 0 | 6.42 |
| 165.10306 | 0 | 1.51 |
| 165.13911 | 0 | 13.79 |
| 166.0983 | 0 | 2.16 |
| 166.12164 | 0 | 20.69 |
| 167.09941 | 0 | 3.07 |
| 167.12274 | 0 | 4.72 |
| 167.15321 | 0 | 0 |
| 168.06691 | 0.01 | 1.3 |
| 168.10852 | 0 | 1.85 |
| 169.0986 | 0 | 1.36 |
| 170.08202 | 0 | 2.46 |
| 170.16531 | 0 | 8.58 |
| 171.11403 | 0 | 2.76 |
| 172.09727 | 0.01 | 1.26 |
| 172.13382 | 0 | 2.77 |
| 174.11226 | 0 | 1.77 |
| 175.14474 | 0 | 17.58 |
| 176.07216 | 0.01 | 1.33 |
| 176.1114 | 0 | 2.44 |
| 177.10342 | 0 | 1.5 |
| 177.13918 | 0 | 32.04 |
| 178.09343 | 0 | 2.2 |
| 179.08113 | 0 | 2.4 |
| 179.11923 | 0 | 1.62 |
| 179.15468 | 0 | 14.56 |
| 180.14972 | 0 | 4.49 |
| 181.09705 | 0 | 1.59 |
| 181.13521 | 0 | 4.26 |
| 182.11818 | 0 | 2.13 |
| 182.1649 | 0 | 43.32 |
| 183.11366 | 0 | 2 |
| 184.10133 | 0 | 1.56 |
| 185.09293 | 0 | 1.35 |
| 185.12938 | 0 | 3.79 |
| 186.07719 | 0 | 9.11 |
| 187.11551 | 0 | 4.02 |
| 188.093 | 0 | 1.47 |
| 190.09579 | 0 | 1.35 |
| 191.12018 | 0 | 2.25 |
| 193.13501 | 0 | 6.5 |
| 193.17024 | 0 | 37.99 |
| 194.08365 | 0 | 2.91 |
| 194.12831 | 0 | 2.13 |
| 194.16614 | 0 | 0 |
| 195.11111 | 0 | 2.9 |
| 195.1593 | 0 | 27.94 |
| 196.1058 | 0 | 1.97 |
| 196.14178 | 0 | 9.07 |
| 197.1297 | 0 | 2.66 |
| 197.16603 | 0.01 | 0 |
| 198.11137 | 0 | 1.76 |
| 198.12729 | 0 | 2.61 |
| 199.11046 | 0 | 1.64 |
| 199.14403 | 0 | 4.85 |
| 200.09294 | 0 | 2.62 |
| 200.1088 | 0 | 3.04 |
| 200.13037 | 0 | 1.74 |
| 201.12563 | 0 | 3.23 |
| 202.10587 | 0 | 1.94 |
| 203.12552 | 0 | 2.31 |
| 205.08813 | 0.01 | 1.52 |
| 206.08292 | 0 | 1.83 |
| 206.13966 | 0 | 1.82 |
| 207.11488 | 0 | 1.66 |
| 207.1855 | 0 | 44.85 |
| 208.1087 | 0.01 | 1.32 |
| 208.14337 | 0 | 14.99 |
| 208.17963 | 0 | 0 |
| 209.12538 | 0 | 1.58 |
| 209.17598 | 0 | 21.74 |
| 210.12006 | 0 | 1.37 |
| 211.11011 | 0 | 1.93 |
| 211.14207 | 0 | 6.36 |
| 212.09253 | 0 | 1.94 |
| 212.13774 | 0 | 14.13 |
| 213.13493 | 0 | 2.91 |
| 213.1581 | 0 | 20.84 |
| 214.10902 | 0 | 2.69 |
| 214.12411 | 0 | 2 |
| 214.13918 | 0 | 1.86 |
| 215.13266 | 0 | 4.15 |
| 216.12392 | 0.01 | 1.32 |
| 217.11235 | 0.02 | 2.02 |
| 217.13446 | 0 | 3.92 |
| 218.08522 | 0 | 8.06 |
| 218.10136 | 0 | 5.77 |
| 218.13229 | 0 | 2.53 |
| 220.10414 | 0 | 2.61 |
| 221.13509 | 0 | 1.61 |
| 222.12608 | 0 | 1.39 |
| 222.16687 | 0 | 24.54 |
| 223.14476 | 0 | 3.04 |
| 224.10144 | 0 | 1.88 |
| 224.1283 | 0 | 1.71 |
| 225.12399 | 0 | 1.61 |
| 225.15858 | 0 | 5.05 |
| 226.11296 | 0 | 2.13 |
| 227.14291 | 0 | 4.43 |
| 229.13916 | 0 | 8.39 |
| 230.10338 | 0 | 1.61 |
| 230.11917 | 0 | 5.07 |
| 231.11523 | 0.02 | 1.27 |
| 232.11569 | 0 | 2.87 |
| 232.15186 | 0 | 2.17 |
| 233.13004 | 0 | 1.72 |
| 234.12694 | 0.01 | 1.2 |
| 235.15248 | 0 | 3.68 |
| 236.09341 | 0 | 3.09 |
| 236.14847 | 0 | 2.04 |
| 237.09802 | 0 | 98.01 |
| 237.15828 | 0 | 0 |
| 238.11804 | 0 | 3.23 |
| 239.12556 | 0 | 2.44 |
| 239.17581 | 0 | 6.13 |
| 240.12663 | 0 | 1.69 |
| 240.14348 | 0 | 1.83 |
| 241.14641 | 0 | 1.68 |
| 241.18842 | 0 | 51 |
| 242.10355 | 0 | 5.5 |
| 242.13145 | 0 | 2.11 |
| 243.1535 | 0 | 48.78 |
| 244.11313 | 0 | 2.71 |
| 244.13339 | 0 | 3.81 |
| 245.13159 | 0 | 1.53 |
| 246.12401 | 0 | 1.37 |
| 246.14056 | 0.02 | 1.63 |
| 247.08327 | 0 | 0 |
| 247.1459 | 0 | 3.49 |
| 248.11443 | 0 | 3.94 |
| 248.14349 | 0 | 5.84 |
| 249.16698 | 0 | 8.4 |
| 250.11577 | 0 | 3.8 |
| 250.14401 | 0 | 4.38 |
| 250.16574 | 0 | 17.67 |
| 251.14235 | 0 | 1.61 |
| 251.1731 | 0 | 0 |
| 252.13632 | 0.01 | 1.29 |
| 253.07124 | 0 | 25.7 |
| 253.15323 | 0 | 6.31 |
| 254.10374 | 0 | 3.04 |
| 255.0979 | 0 | 92.94 |
| 255.16194 | 0 | 0 |
| 256.12964 | 0 | 2.58 |
| 257.14365 | 0 | 1.59 |
| 257.16467 | 0 | 5.27 |
| 258.12747 | 0 | 1.7 |
| 258.15234 | 0 | 6.01 |
| 259.14651 | 0 | 3.43 |
| 260.11591 | 0 | 26.6 |
| 260.13885 | 0.05 | 1.27 |
| 261.16412 | 0 | 9.1 |
| 262.12027 | 0 | 6.7 |
| 262.1553 | 0 | 4.55 |
| 263.14795 | 0.04 | 1.27 |
| 263.17371 | 0 | 104.31 |
| 264.13437 | 0 | 1.4 |
| 264.16678 | 0 | 0 |
| 265.17737 | 0 | 1.9 |
| 266.11847 | 0 | 2.3 |
| 266.15305 | 0.03 | 3.29 |
| 266.16861 | 0 | 3.34 |
| 268.1041 | 0.01 | 1.76 |
| 269.11493 | 0 | 3.04 |
| 269.15939 | 0 | 7.1 |
| 269.18301 | 0 | 6.79 |
| 270.12454 | 0 | 2.43 |
| 271.08231 | 0 | 13.93 |
| 271.14587 | 0.03 | 1.64 |
| 271.17276 | 0 | 11.36 |
| 272.11258 | 0 | 8.47 |
| 273.14459 | 0.03 | 1.68 |
| 273.16095 | 0 | 14.53 |
| 274.12677 | 0 | 6.6 |
| 274.16327 | 0 | 21.25 |
| 275.14789 | 0.01 | 1.53 |
| 275.1749 | 0 | 0 |
| 276.14532 | 0 | 2.15 |
| 276.17273 | 0 | 22.21 |
| 278.1225 | 0 | 0 |
| 279.13705 | 0 | 5.13 |
| 279.17844 | 0 | 6.54 |
| 280.13037 | 0 | 4.85 |
| 280.15686 | 0.03 | 2.03 |
| 280.17358 | 0 | 3.48 |
| 281.17715 | 0 | 11.51 |
| 282.14368 | 0 | 1.51 |
| 285.1524 | 0.03 | 1.85 |
| 285.18057 | 0 | 90.17 |
| 286.13641 | 0 | 4.11 |
| 287.16721 | 0 | 21.12 |
| 288.12396 | 0 | 12.42 |
| 288.16541 | 0 | 13.23 |
| 289.0925 | 0 | 12.87 |
| 289.18735 | 0 | 19.49 |
| 290.12054 | 0 | 24.59 |
| 290.19034 | 0 | 0 |
| 291.18463 | 0 | 3.23 |
| 292.14029 | 0 | 3.33 |
| 292.17502 | 0 | 11.61 |
| 293.18167 | 0 | 0 |
| 294.14542 | 0 | 2 |
| 298.14438 | 0 | 2.61 |
| 299.15405 | 0.05 | 1.4 |
| 299.1936 | 0 | 33.71 |
| 300.14804 | 0 | 2.54 |
| 301.16559 | 0 | 1.49 |
| 303.17712 | 0 | 2.15 |
| 303.19531 | 0 | 3.2 |
| 304.15576 | 0.04 | 2.21 |
| 304.1915 | 0 | 9.53 |
| 305.16501 | 0.04 | 1.25 |
| 305.18427 | 0 | 22.58 |
| 306.15262 | 0 | 2.4 |
| 306.19528 | 0 | 19.47 |
| 307.18753 | 0 | 1.9 |
| 308.20087 | 0 | 11.3 |
| 309.14856 | 0.01 | 1.93 |
| 309.19034 | 0 | 5.55 |
| 310.15112 | 0 | 3.38 |
| 315.18857 | 0 | 2.46 |
| 317.16428 | 0 | 1.49 |
| 317.21393 | 0 | 123.23 |
| 318.15991 | 0 | 3.18 |
| 321.18704 | 0 | 1.5 |
| 329.16632 | 0 | 1.78 |
| 330.16391 | 0 | 2.22 |
| 331.18277 | 0 | 1.65 |
| 332.17786 | 0 | 2.67 |
| 333.19409 | 0 | 1.59 |
| 345.19199 | 0 | 1.61 |
| 347.17624 | 0 | 2.37 |
| 348.17508 | 0 | 4.83 |
| 349.19281 | 0 | 1.53 |
| 373.19687 | 0 | 1.86 |
| 482.30594 | 0.04 | 1.84 |
| 526.33179 | 0.05 | 2.04 |
| 570.3576 | 0.05 | 2.19 |


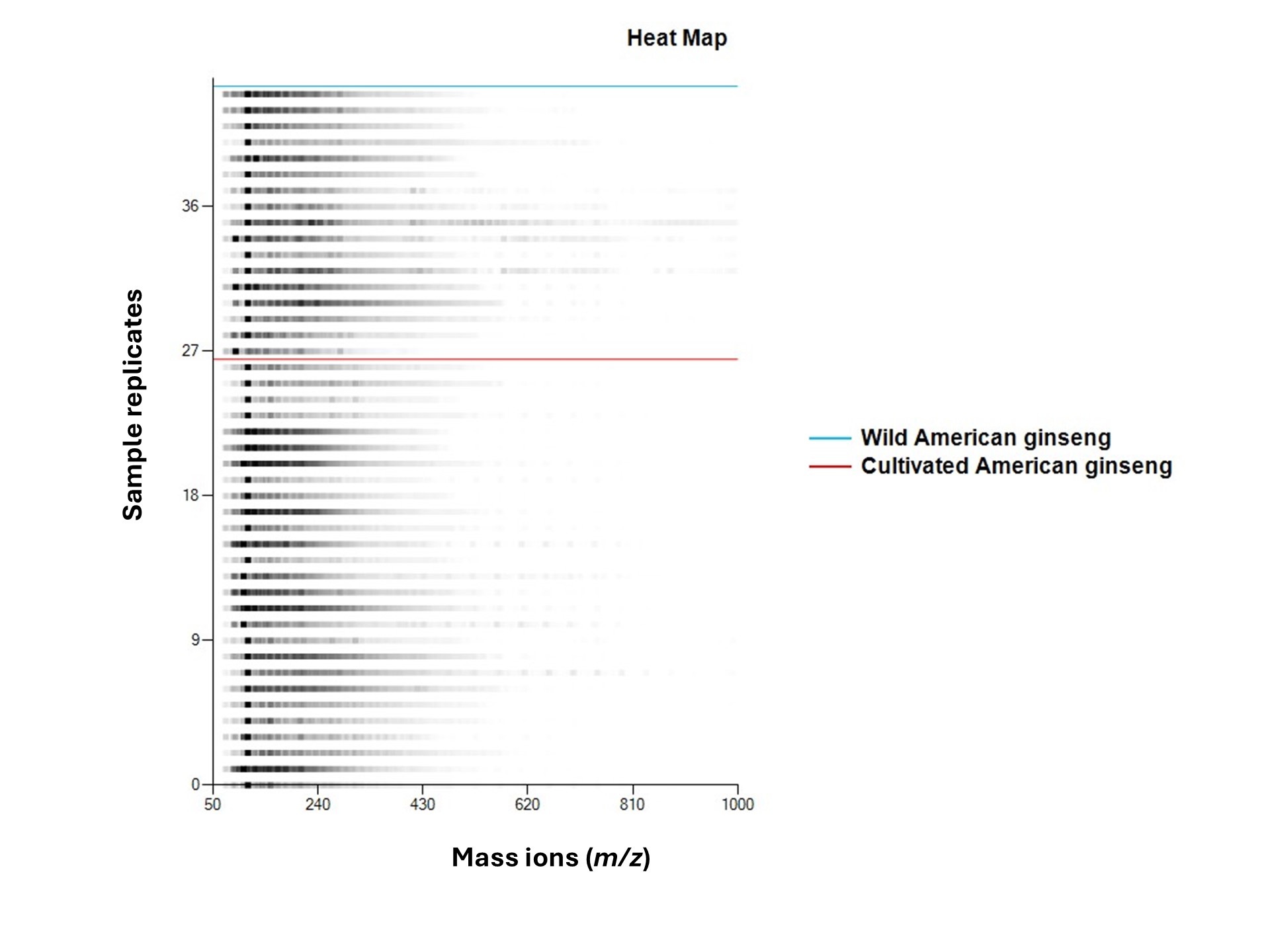
**Figure S6:** Heat map of mass ions observed during step 2 (provenance) classification model.

**Table S6:** List of selected ions used to build step 2 PCA and DAPC. The fold values refer to the difference in mean abundance for that m/z between the two classes and the p value from analysis by ANOVA.

| **Selected m/z's** | **p** | **Fold** |
| --- | --- | --- |
| 81.03397 | 0.01 | 2.6 |
| 87.04418 | 0.04 | 3.91 |
| 88.07575 | 0.02 | 2.01 |
| 89.10751 | 0.01 | 9.93 |
| 90.05489 | 0.01 | 2.45 |
| 90.09132 | 0.01 | 7.96 |
| 91.09444 | 0.01 | 8.91 |
| 97.07553 | 0.03 | 1.36 |
| 98.05936 | 0.02 | 1.79 |
| 103.0866 | 0.01 | 2.72 |
| 104.0705 | 0 | 2.83 |
| 105.0735 | 0 | 3.39 |
| 108.0444 | 0 | 8.79 |
| 109.0761 | 0 | 1.99 |
| 110.0599 | 0 | 1.6 |
| 111.0443 | 0 | 1.68 |
| 111.0917 | 0 | 2.05 |
| 115.051 | 0 | 3.24 |
| 123.0914 | 0 | 2.87 |
| 124.0754 | 0.01 | 1.82 |
| 124.1525 | 0.01 | 3.01 |
| 125.1071 | 0 | 2.02 |
| 126.0548 | 0 | 3.39 |
| 126.1027 | 0 | 1.77 |
| 128.075 | 0 | 1.73 |
| 129.0662 | 0 | 2.35 |
| 133.0744 | 0.03 | 1.64 |
| 137.0816 | 0 | 2.71 |
| 137.1072 | 0 | 2.1 |
| 138.1029 | 0 | 1.76 |
| 139.1227 | 0 | 2.31 |
| 140.071 | 0 | 1.64 |
| 140.118 | 0 | 1.73 |
| 141.1027 | 0 | 1.95 |
| 143.0845 | 0.02 | 2.03 |
| 144.066 | 0.05 | 1.48 |
| 146.1647 | 0.02 | 3.2 |
| 149.1073 | 0 | 3 |
| 151.0856 | 0 | 18.19 |
| 151.1234 | 0 | 1.96 |
| 152.1178 | 0 | 1.87 |
| 153.1025 | 0 | 1.63 |
| 153.1385 | 0 | 2.16 |
| 155.1182 | 0 | 2.88 |
| 158.082 | 0.01 | 1.73 |
| 160.0968 | 0.01 | 1.55 |
| 161.1066 | 0 | 1.46 |
| 162.0911 | 0 | 1.57 |
| 163.1233 | 0 | 1.88 |
| 165.1391 | 0 | 2.52 |
| 166.0979 | 0 | 1.69 |
| 166.1216 | 0 | 4.63 |
| 167.0952 | 0 | 2.13 |
| 167.1227 | 0 | 1.48 |
| 170.1653 | 0 | 4.87 |
| 172.1339 | 0.01 | 2.07 |
| 173.1335 | 0.03 | 1.89 |
| 174.1123 | 0.01 | 1.44 |
| 177.1034 | 0.03 | 1.39 |
| 177.1392 | 0 | 2.29 |
| 178.0935 | 0 | 1.62 |
| 179.0811 | 0 | 13.36 |
| 179.1547 | 0 | 2.4 |
| 180.1054 | 0.01 | 1.71 |
| 180.1497 | 0 | 1.96 |
| 181.1352 | 0 | 1.71 |
| 182.1649 | 0 | 3.6 |
| 183.1137 | 0 | 1.27 |
| 183.1504 | 0 | 2.57 |
| 185.0929 | 0 | 1.75 |
| 185.1294 | 0 | 1.67 |
| 186.1102 | 0 | 1.52 |
| 190.0958 | 0 | 1.49 |
| 191.1534 | 0 | 2.83 |
| 192.1117 | 0 | 1.48 |
| 193.0992 | 0 | 1.88 |
| 193.135 | 0.02 | 1.28 |
| 193.1702 | 0 | 3.28 |
| 194.0837 | 0 | 2.86 |
| 194.1661 | 0 | 4.71 |
| 196.1058 | 0 | 1.77 |
| 196.1418 | 0.02 | 1.57 |
| 197.0973 | 0 | 12.85 |
| 197.1297 | 0 | 1.49 |
| 197.166 | 0.04 | 0 |
| 198.1114 | 0.04 | 1.48 |
| 199.1105 | 0.02 | 1.4 |
| 199.144 | 0.01 | 1.78 |
| 201.0996 | 0 | 0 |
| 201.1256 | 0.03 | 1.26 |
| 202.108 | 0.01 | 1.38 |
| 203.1255 | 0.05 | 1.16 |
| 203.1804 | 0 | 48.11 |
| 204.1839 | 0 | 0 |
| 205.0881 | 0 | 2.65 |
| 205.1687 | 0 | 3 |
| 205.1957 | 0.01 | 0 |
| 206.0829 | 0 | 1.99 |
| 207.1855 | 0 | 3.28 |
| 208.1085 | 0.01 | 1.5 |
| 208.1434 | 0 | 5.09 |
| 208.1796 | 0 | 2.33 |
| 209.176 | 0.04 | 1.49 |
| 210.1201 | 0 | 1.35 |
| 211.1101 | 0 | 6.84 |
| 211.1421 | 0 | 1.64 |
| 212.0925 | 0 | 2.01 |
| 212.1377 | 0.03 | 1.51 |
| 214.1392 | 0.02 | 1.56 |
| 215.1327 | 0 | 1.48 |
| 216.1239 | 0.04 | 1.31 |
| 217.1124 | 0 | 17.99 |
| 217.1345 | 0 | 1.44 |
| 219.1141 | 0 | 1.83 |
| 220.1046 | 0 | 1.85 |
| 220.1422 | 0 | 3.2 |
| 222.1261 | 0 | 1.47 |
| 222.1669 | 0 | 7.65 |
| 224.1014 | 0 | 2.59 |
| 225.1586 | 0.01 | 1.51 |
| 226.113 | 0 | 3.46 |
| 229.1185 | 0 | 13.52 |
| 229.1392 | 0 | 1.95 |
| 230.1044 | 0 | 3.2 |
| 231.1152 | 0 | 2.41 |
| 231.1479 | 0 | 2.79 |
| 232.1115 | 0 | 2.43 |
| 232.1519 | 0.01 | 1.34 |
| 233.166 | 0.04 | 2.6 |
| 235.1525 | 0.01 | 1.57 |
| 236.0925 | 0 | 3.16 |
| 236.1485 | 0.02 | 1.34 |
| 237.1243 | 0 | 1.93 |
| 237.1583 | 0 | 1.96 |
| 238.1162 | 0 | 2.12 |
| 239.1256 | 0 | 2.44 |
| 240.1266 | 0 | 2.4 |
| 240.1435 | 0 | 2.76 |
| 241.1464 | 0 | 1.8 |
| 241.1884 | 0.02 | 2.59 |
| 242.1315 | 0.01 | 1.85 |
| 243.1283 | 0 | 7.29 |
| 244.1131 | 0 | 7.77 |
| 244.154 | 0.02 | 2.26 |
| 245.1892 | 0.03 | 3.65 |
| 246.124 | 0 | 1.66 |
| 247.1459 | 0 | 1.43 |
| 248.1254 | 0.01 | 2.08 |
| 249.1305 | 0.04 | 1.49 |
| 249.167 | 0.01 | 1.86 |
| 250.1244 | 0 | 4.46 |
| 250.1657 | 0.02 | 1.96 |
| 251.1731 | 0.01 | 3.88 |
| 252.1363 | 0.01 | 1.53 |
| 252.1518 | 0 | 3.15 |
| 254.1037 | 0 | 3.7 |
| 255.134 | 0 | 2.34 |
| 255.1619 | 0 | 2.41 |
| 256.1281 | 0 | 2.27 |
| 257.1437 | 0 | 2.58 |
| 258.1275 | 0 | 3.7 |
| 260.1389 | 0 | 1.46 |
| 261.1641 | 0 | 1.91 |
| 262.1553 | 0.02 | 1.94 |
| 263.148 | 0 | 1.71 |
| 263.1737 | 0.01 | 2.62 |
| 264.1344 | 0 | 1.98 |
| 264.1668 | 0 | 4.28 |
| 265.1774 | 0.03 | 1.68 |
| 266.1185 | 0.01 | 2.49 |
| 266.1686 | 0 | 2.38 |
| 268.1049 | 0 | 3.49 |
| 269.1149 | 0 | 19.64 |
| 269.1427 | 0 | 4.18 |
| 270.1245 | 0 | 4.06 |
| 270.1679 | 0 | 6.69 |
| 272.1314 | 0 | 3.07 |
| 273.1446 | 0.03 | 1.91 |
| 273.161 | 0 | 1.86 |
| 274.1366 | 0 | 6.41 |
| 274.1633 | 0 | 2.06 |
| 275.1479 | 0 | 3.16 |
| 275.1749 | 0 | 2.98 |
| 276.1453 | 0 | 5.9 |
| 276.1727 | 0 | 3.83 |
| 278.1506 | 0 | 7.57 |
| 278.1751 | 0 | 2.38 |
| 278.2105 | 0.04 | 3.7 |
| 279.1597 | 0.01 | 2.88 |
| 279.1784 | 0.01 | 2.58 |
| 279.2325 | 0 | 4.54 |
| 280.1304 | 0.01 | 5.1 |
| 280.1569 | 0.03 | 2.07 |
| 281.1495 | 0 | 2.46 |
| 281.1772 | 0 | 2.54 |
| 281.2479 | 0 | 3.57 |
| 282.1437 | 0 | 2.72 |
| 284.14 | 0 | 2.4 |
| 285.1524 | 0.04 | 2.02 |
| 286.1364 | 0.01 | 4.44 |
| 287.1499 | 0.01 | 1.66 |
| 287.1672 | 0 | 2.46 |
| 288.1473 | 0 | 3.43 |
| 288.1654 | 0 | 2.42 |
| 289.1582 | 0 | 3.21 |
| 289.1874 | 0 | 4.68 |
| 290.1481 | 0 | 6.48 |
| 290.1903 | 0 | 3.46 |
| 291.1548 | 0.01 | 8.12 |
| 291.1846 | 0.01 | 1.64 |
| 292.1403 | 0 | 6.28 |
| 292.175 | 0 | 2.45 |
| 294.1454 | 0 | 4.02 |
| 294.1707 | 0 | 4.12 |
| 295.1501 | 0 | 0 |
| 295.197 | 0.03 | 5.76 |
| 296.1501 | 0 | 10.84 |
| 297.1548 | 0 | 4.9 |
| 298.1444 | 0 | 57.12 |
| 299.1541 | 0.01 | 2.51 |
| 300.148 | 0 | 4.66 |
| 302.1637 | 0 | 4.55 |
| 302.1854 | 0 | 2.5 |
| 303.1953 | 0 | 2.19 |
| 304.1558 | 0 | 6.09 |
| 304.1915 | 0 | 3.51 |
| 305.165 | 0 | 2.42 |
| 306.1526 | 0 | 13.45 |
| 306.1953 | 0 | 2.9 |
| 308.1718 | 0.01 | 3 |
| 308.2009 | 0.03 | 2.45 |
| 309.1486 | 0 | 17.19 |
| 310.1511 | 0 | 30.72 |
| 314.1596 | 0 | 8.98 |
| 315.1716 | 0.01 | 1.89 |
| 315.1886 | 0.01 | 1.63 |
| 316.1526 | 0 | 0 |
| 317.1643 | 0.04 | 1.65 |
| 317.2139 | 0 | 21.28 |
| 318.1599 | 0 | 0 |
| 318.2048 | 0 | 3.38 |
| 320.1839 | 0 | 2.2 |
| 320.2027 | 0 | 3.24 |
| 322.1627 | 0 | 8.51 |
| 323.1679 | 0 | 4.11 |
| 323.1916 | 0.05 | 1.65 |
| 326.1601 | 0 | 0 |
| 329.1663 | 0.01 | 1.89 |
| 330.1639 | 0 | 6.35 |
| 332.1779 | 0 | 12.75 |
| 332.2083 | 0.01 | 2.28 |
| 335.1781 | 0 | 1.98 |
| 342.1675 | 0 | 0 |
| 343.1795 | 0 | 2.4 |
| 344.1779 | 0.02 | 3.23 |
| 347.1762 | 0 | 2.71 |
| 348.1751 | 0 | 0 |
| 350.186 | 0 | 3.77 |
| 359.1884 | 0 | 4.81 |
| 360.1842 | 0 | 21.32 |
| 362.1971 | 0 | 11.04 |
| 365.187 | 0 | 2.36 |
| 373.1969 | 0 | 2.15 |
| 411.3987 | 0.02 | 5.17 |
| 412.4022 | 0.02 | 5.19 |
| 423.3625 | 0.01 | 0 |
| 428.4253 | 0.02 | 4.29 |
| 441.3727 | 0.01 | 0 |
| 575.5041 | 0.01 | 112.06 |


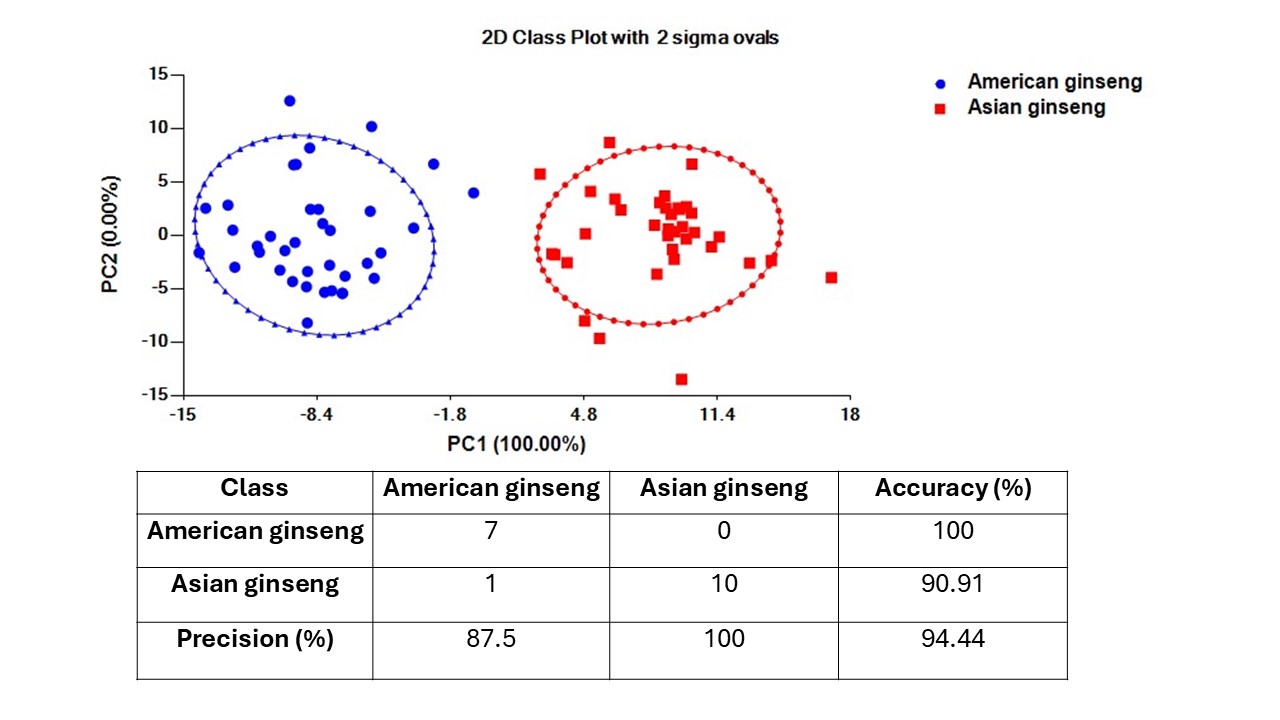


**Figure S7**: External validation results based on step 1 DAPC Model. The 2 sigma ovals represent the 95% confidence limits encircling each class.


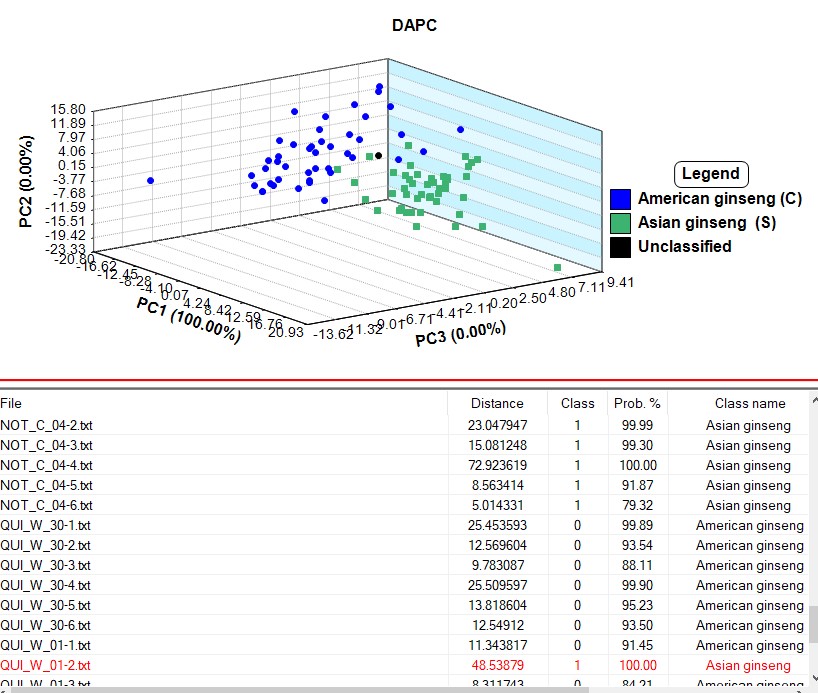


**Figure S8**: View of a portion of results of Leave one out cross validation (LOOCV) for step 1 DAPC model below the DAPC scatter plot. Two Chinese ginseng (*P. notoginseng*) and one wild American ginseng (*P. quinquefolius*) were misassigned as American and Asian ginseng respectively yielding a score of 96.74%.


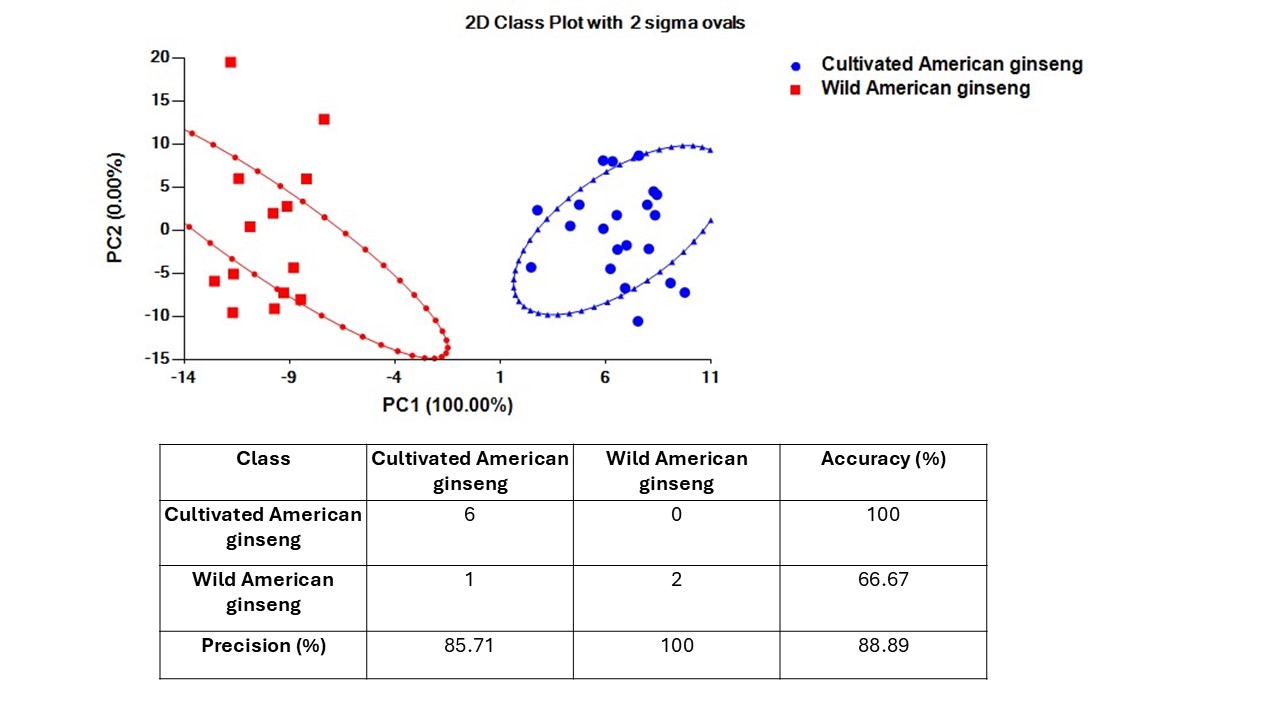


**Figure S9:** External validation results step 2 DAPC Model. The 2 sigma ovals represent the 95% confidence limits encircling each class.


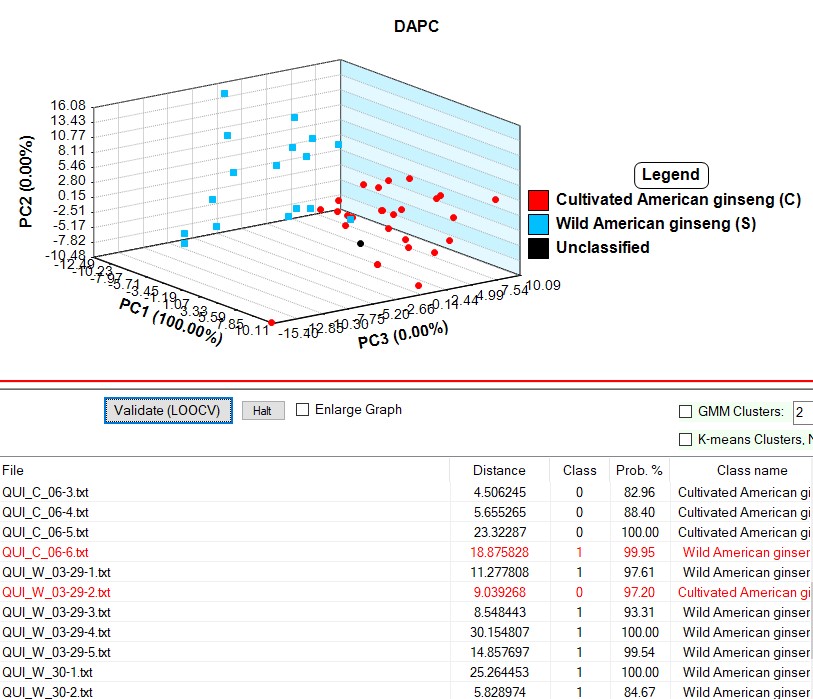


**Figure S10:** View of a portion of results of Leave one out cross validation (LOOCV) for step 2 DAPC model below the DAPC scatter plot. Three cultivated American ginseng samples were misclassified as wild American ginseng. Three wild American ginseng samples were misclassified as cultivated American ginseng. This yielded a LOOCV score of 86.36%.


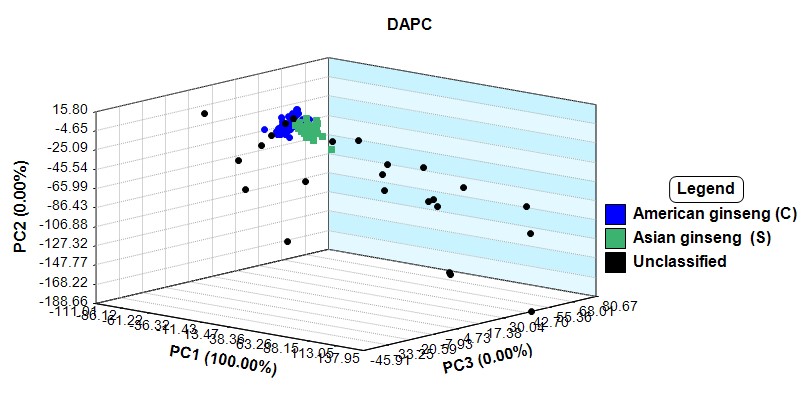


**Figure S11:** Step 1 DAPC classification results for QA samples plot.

**Table S7:** Step 1 (species) DAPC classification results for QA samples.

| **File** | **Distance** | **Class** | **Prob. %** | **Class name** | **Known Class Name** |
| --- | --- | --- | --- | --- | --- |
| QA 1 - 1.txt | 131.1779 | 0 | 100 | American ginseng | Unknown |
| QA 1 - 2.txt | 210.3504 | 0 | 100 | American ginseng | Unknown |
| QA 1 - 3.txt | 22.06177 | 1 | 99.98 | Asian ginseng | Unknown |
| QA 1 - 4.txt | 95.34458 | 0 | 100 | American ginseng | Unknown |
| QA 1 - 5.txt | 102.8799 | 0 | 100 | American ginseng | Unknown |
| QA 1 - 6.txt | 217.4967 | 0 | 100 | American ginseng | Unknown |
| QA 2 - 1.txt | 208.3145 | 1 | 100 | Asian ginseng | Unknown |
| QA 2 - 2.txt | 136.2258 | 1 | 100 | Asian ginseng | Unknown |
| QA 2 - 3.txt | 190.9951 | 1 | 100 | Asian ginseng | Unknown |
| QA 2 - 4.txt | 143.5089 | 1 | 100 | Asian ginseng | Unknown |
| QA 2 - 5.txt | 98.44174 | 1 | 100 | Asian ginseng | Unknown |
| QA 2 - 6.txt | 78.3054 | 1 | 100 | Asian ginseng | Unknown |
| QA 3 - 1.txt | 242.8121 | 0 | 100 | American ginseng | Unknown |
| QA 3 - 2.txt | 85.95386 | 0 | 100 | American ginseng | Unknown |
| QA 3 - 3.txt | 128.6118 | 0 | 100 | American ginseng | Unknown |
| QA 3 - 4.txt | 80.43368 | 0 | 100 | American ginseng | Unknown |
| QA 3 - 5.txt | 195.4975 | 0 | 100 | American ginseng | Unknown |
| QA 3 - 6.txt | 92.53565 | 0 | 100 | American ginseng | Unknown |
| QA 4 - 1.txt | 45.09623 | 1 | 100 | Asian ginseng | Unknown |
| QA 4 - 2.txt | 70.71166 | 1 | 100 | Asian ginseng | Unknown |
| QA 4 - 3.txt | 49.69095 | 1 | 100 | Asian ginseng | Unknown |
| QA 4 - 4.txt | 17.07505 | 1 | 99.73 | Asian ginseng | Unknown |
| QA 4 - 5.txt | 138.2883 | 0 | 100 | American ginseng | Unknown |
| QA 4 - 6.txt | 213.1043 | 1 | 100 | Asian ginseng | Unknown |


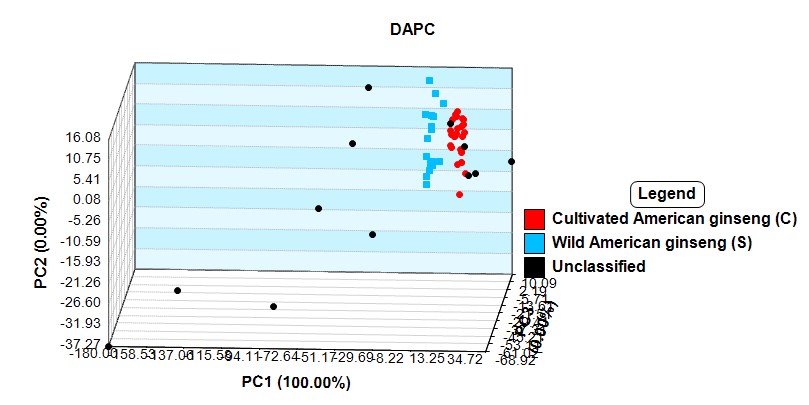


**Figure S12:** Step 2 DAPC classification results for QA samples plot.

**Table S8:** Step 2 (provenance) DAPC classification results for QA samples.


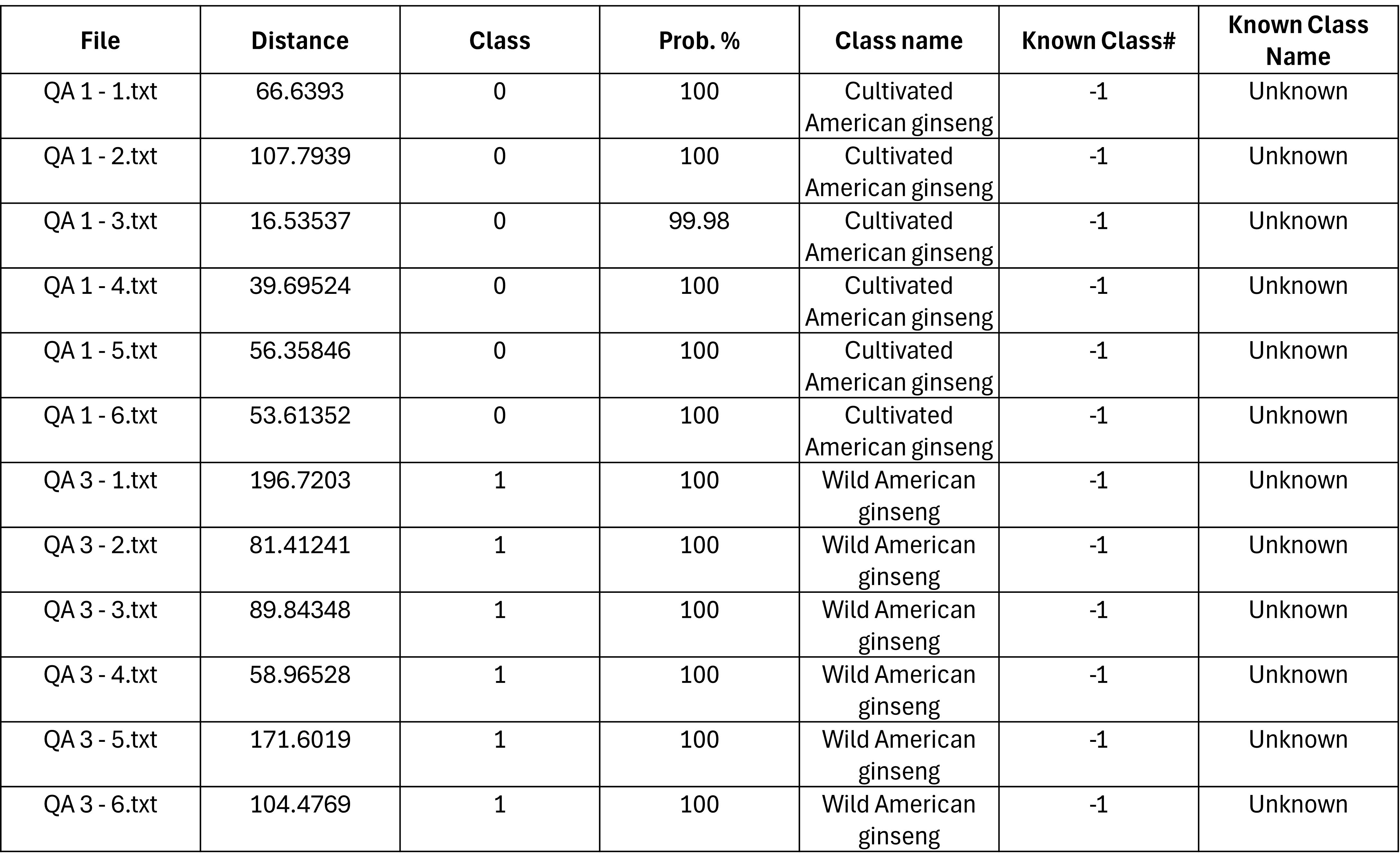

Supplement: Supplementary file 1 — Additional file 1. [file 40068_2025_414_MOESM1_ESM.docx]
